# Diversification of habenular organization and asymmetries in teleosts: Insights from the Atlantic salmon and European eel

**DOI:** 10.3389/fcell.2022.1015074

**Published:** 2022-11-03

**Authors:** Léo Michel, Karina Palma, Mauricio Cerda, Ronan Lagadec, Hélène Mayeur, Michaël Fuentès, Laurence Besseau, Patrick Martin, Elodie Magnanou, Patrick Blader, Miguel L. Concha, Sylvie Mazan

**Affiliations:** ^1^ UMR7232-Biologie Intégrative des Organismes Marins (BIOM), Observatoire Océanologique, CNRS-Sorbonne Université, Banyuls sur Mer, France; ^2^ Integrative Biology Program, Institute of Biomedical Sciences, Facultad de Medicina. Universidad de Chile, Santiago, Chile; ^3^ Biomedical Neuroscience Institute, Santiago, Chile; ^4^ Center for Medical Informatics and Telemedicine, Facultad de Medicina, Santiago, Chile; ^5^ Conservatoire National du Saumon Sauvage, Chanteuges, France; ^6^ Molecular Cellular and Developmental Biology (MCD UMR5077), Centre de Biologie Intégrative (CBI FR 3743), CNRS, UPS, Université de Toulouse, Toulouse, France; ^7^ Geroscience Center for Brain Health and Metabolism, Santiago, Chile

**Keywords:** habenula, asymmetry, Atlantic salmon, European eel, pax6, sox1

## Abstract

Habenulae asymmetries are widespread across vertebrates and analyses in zebrafish, the reference model organism for this process, have provided insight into their molecular nature, their mechanisms of formation and their important roles in the integration of environmental and internal cues with a variety of organismal adaptive responses. However, the generality of the characteristics identified in this species remains an open question, even on a relatively short evolutionary scale, in teleosts. To address this question, we have characterized the broad organization of habenulae in the Atlantic salmon and quantified the asymmetries in each of the identified subdomains. Our results show that a highly conserved partitioning into a dorsal and a ventral component is retained in the Atlantic salmon and that asymmetries are mainly observed in the former as in zebrafish. A remarkable difference is that a prominent left-restricted pax6 positive nucleus is observed in the Atlantic salmon, but undetectable in zebrafish. This nucleus is not observed outside teleosts, and harbors a complex presence/absence pattern in this group, retaining its location and cytoarchitectonic organization in an elopomorph, the European eel. These findings suggest an ancient origin and high evolvability of this trait in the taxon. Taken together, our data raise novel questions about the variability of asymmetries across teleosts and their biological significance depending on ecological contexts.

## 1 Introduction

Habenulae are a bilateral epithalamic structure, which are present in all vertebrates and form a relay in highly conserved neuronal circuits connecting multiple forebrain nuclei to midbrain or hindbrain centers, such as the ventral tegmental area, interpeduncular nucleus or raphe nuclei (Bianco and Wilson, 2009; Fakhoury, 2018; Hu et al., 2020). They are thought to act as a switchboard, which integrates sensory inputs and internal states to modulate a variety of behavioral, emotional and cognitive processes, including responses to threats or aversives, social interactions or aversive learning (Fore et al., 2018). Habenulae can be subdivided into two evolutionarily conserved subdivisions (respectively medial and lateral in the mouse, dorsal and ventral in the zebrafish (Amo et al., 2010). These two components are distinguished by conserved projection patterns and shared molecular signatures, but in line with the complexity of their functions, they consist of heterogeneous neuronal subpopulations, whose relationships across these distant species remain elusive (Pandey et al., 2018; Hashikawa et al., 2020; Wallace et al., 2020). A remarkable specificity of this epithalamic structure is that it displays asymmetries in size, cytoarchitecture, patterns of projections and neuronal identities, which considerably vary in degree across vertebrates [reviewed in (Concha and Wilson, 2001)]. Thus far, their molecular nature and their roles have been essentially described in the zebrafish, the reference model organism for this process. In this species, asymmetries are restricted to the dorsal habenula (dHb) and consist of different relative proportions between two nuclei, an early-born lateral one (dHbl) and a late-born medial one (dHbm), which prevail respectively on the left and the right side (Gamse et al., 2005; Aizawa et al., 2007; deCarvalho et al., 2014). Asymmetries in dHb result in differential processing of sensory signals between the left and the right habenula sides (Dreosti et al., 2014) and their abolition affects behavioral and emotional responses to environmental cues, including fear responses, food seeking, and light preference (Facchin et al., 2015; Duboué et al., 2017; Zhang et al., 2017; Chen et al., 2019; Choi et al., 2021).

The establishment of habenular asymmetries has been extensively analyzed in the zebrafish, and it requires local interactions with the parapineal organ, a component of the midline-derived pineal complex that in embryonic stages migrates to the left side (Concha et al., 2003; Gamse et al., 2003; Regan et al., 2009). Critically, yet unidentified signals produced by the parapineal, influence the left habenula and result in a transient and left-restricted repression of Wnt signaling. This differential regulation promotes dHbl neuronal fate choice, possibly through a modulation of the timing of neurogenesis (Hüsken et al., 2014; Guglielmi et al., 2020), and it is dependent on the expression of *sox1a* in the parapineal (Lekk et al., 2019). Our extensive knowledge on the nature, roles and mechanisms of formation of habenular asymmetries in the zebrafish contrasts with the paucity of data on their mode of diversification across vertebrates. Analyses of a cartilaginous fish (catshark *Scyliorhinus canicula*) and of a cyclostome (or agnathan, lamprey *Lampetra fluviatilis*) have suggested that the mechanisms employed by the zebrafish for their elaboration may have substantially diverged from the vertebrate ancestral state (Lagadec et al., 2015). But even at a much shorter evolutionary scale, across teleosts, habenula shape, cytoarchitecture and morphological asymmetry exhibit considerable variations, which question mechanistic conservations (Villalón et al., 2012; Signore and Concha, 2017). In order to further assess, and obtain molecular readouts of these variations, we have characterized the broad subdivisions of habenulae in the Atlantic salmon *Salmo salar*. This species indeed harbors significant habenular asymmetries (Eilertsen et al., 2021) and differs substantially from zebrafish by its phylogenetic position in teleosts, its size and a complex life cycle involving a major transition from fresh water to a marine environment (Björnsson et al., 2011). We focused on salmon orthologues of genes sharing expressions in discrete habenula subdomains across gnathostomes: *kctd12b*, *sox1* and *kiss1*, which respectively code for an auxiliary GABA_B_ receptor subunit modulating neurotransmitter release from medial habenula terminals in the mouse (Schwenk et al., 2010; Bhandari et al., 2021), a transcription factor marking the lateral habenula in the mouse (Kan et al., 2007) and a neuropeptide broadly expressed in the ventral habenula and involved in the regulation of fear responses and aversive learning in the zebrafish (Ogawa et al., 2014; Nathan et al., 2015; Lupton et al., 2017). We also included analysis of pax6, a general neural progenitor marker (reviewed in Schuurmans and Guillemot, 2002), in order to characterize their territory and assess the mode of habenula growth during smoltification. Comparisons highlight similarities with zebrafish, but also a notable difference, with the presence of a pax6-positive nucleus restricted to the left side, identified in several teleost lineages but undetectable in the zebrafish. These data show that comparative analyses at different evolutionary scales can be important for a comprehensive view of habenular asymmetries and of their variations across vertebrates.

## 2 Materials and methods

### 2.1 Tissue collection and fixation

Atlantic salmon specimens at different stages of smoltification or lower mode yearlings (that will not smoltify the following year) were obtained from the Conservatoire National du saumon sauvage, Chanteuges, and France. False clownfishes and zebrafish were bred by the Aquariology service, Banyuls sur Mer. Specimens from other species were purchased from professional fishermen (European eel) or aquariology fish suppliers (reedfish, spotted gar, silver and arowana). In all species, brains were collected from euthanized specimens, fixed, dehydrated and stored at −20°C until sectioning for subsequent analyses. Stages were determined after (Roux et al., 2019) for the false clownfish. The status of Atlantic salmon specimens relative to smoltification was assessed by morphology and color changes as described in (McCormick and Saunders, 1987).

### 2.2 Immunohistochemistry of sections

Fixed brains were embedded in paraffin and sectioned (thickness: 6–8 μm). After epitope unmasking, sections were subjected to immunohistochemistry. Fluorescent immunohistochemical analyses of sections were conducted as previously described (Lagadec et al., 2018) using antibodies and concentrations listed in Supplementary Table S1. Brain sections were imaged with a Leica SP8 confocal laser-scanning microscope and images were processed using ImageJ.

### 2.3 3D reconstruction: Image processing and analysis

Confocal images of habenulae from parr lower mode, parr upper mode and smolt specimens were automatically aligned with the Linear Stack Alignment with SIFT FIJI plugin (Lowe, 2004), using the nuclei channel as a reference. Manual segmentation of the left and right habenulae and their subdomains were performed in aligned stacks for each stage, by drawing structure contours in each registered plane using the ROI tool in FIJI (Schindelin et al., 2012). Binary ROIs from this segmentation were used to generate 3D surface mesh visualization and volume quantifications using the SCIAN-Soft, a custom-build software platform programmed in IDL 7.1.2 (ITT/Harris; Boulder, CO, United States).

### 2.4 *In situ* hybridization of sections

ISH of sections were conducted using digoxigenin-labeled antisense RNA probes, transcribed *in vitro* from synthetic gene fragments (Derobert et al., 2002). Probe sequences are listed in Supplementary Table S2. Following ISH, nuclei were counterstained using Nuclear Fast Red (Sigma) and mounted using Eukitt (Sigma). Brain sections were imaged with a Zeiss Axioplan 2 microscope. Brightfield images of ISH for *Sskctd12b*, *Sskiss1* and *Ssox1b* were automatically aligned with the Linear Stack Alignment with SIFT FIJI plugin (Lowe, 2004), using a single channel as reference (the three channels were tested, and best was selected). Then, the color palette of each brightfield image was inverted using the INVERT toll in FIJI (Schindelin et al., 2012), leaving the signal in white and a black background. Subsequently, each aligned image was pseudo-colored using the LOOKUP TABLE tool in FIJI. Finally, the three signals were combined into a single image using the IMAGE CALCULATOR tool of FIJI.

## 3 Results

### 3.1 Dorsal and ventral habenular territories harbor different cell organizations in the Atlantic salmon

**FIGURE 1:**
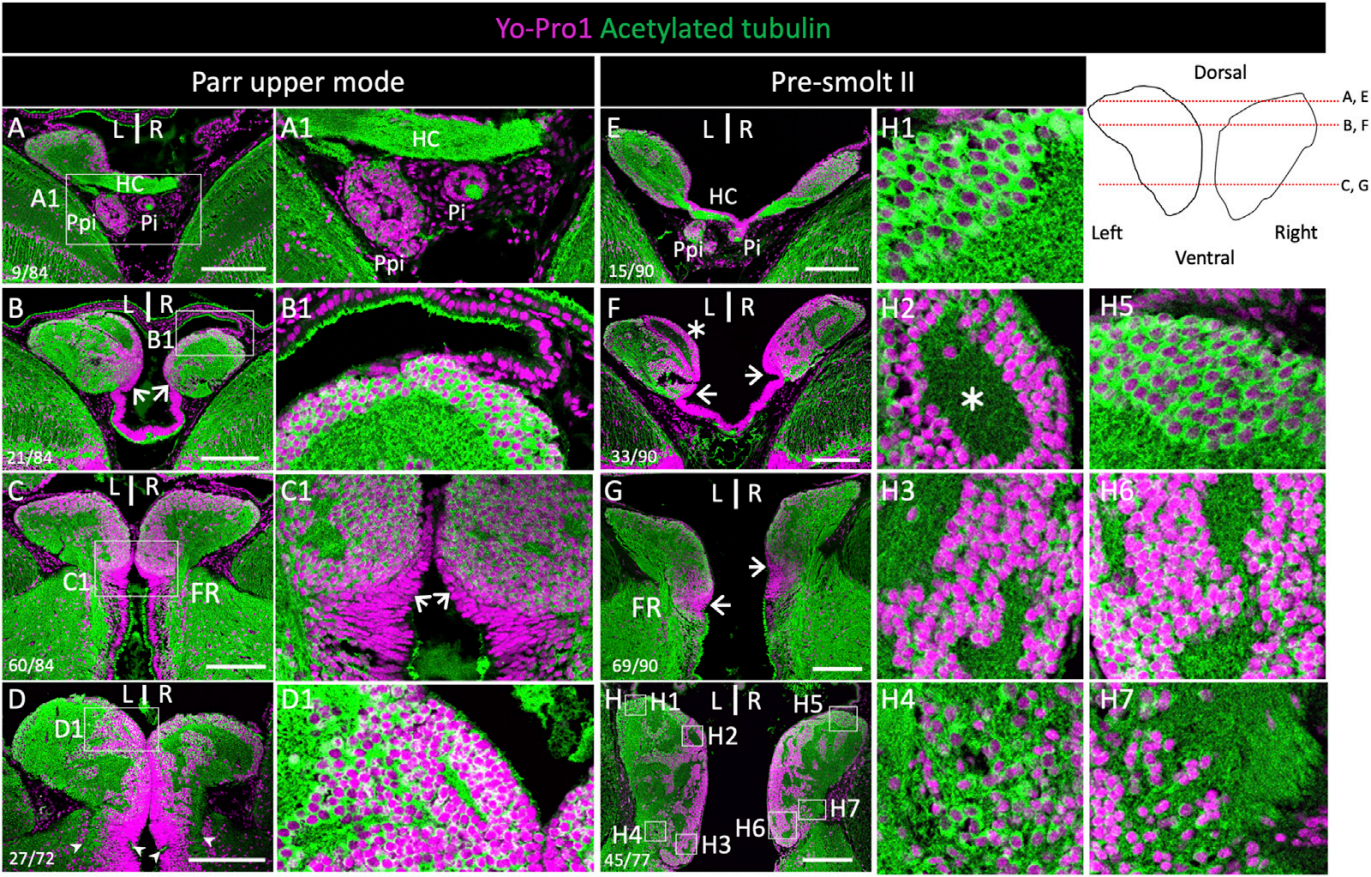
Cytoarchitecture of habenulae in Atlantic salmon during smoltification. **(A–D)** and **(E–H)** show histological sections of the habenulae in parr upper mode and pre-smolt II respectively, following nuclear staining using Yo-Pro1 (purple) and IHC with an antibody directed against acetylated tubulin (green). **(A–G)** are horizontal sections, **(D,H)** are transverse sections. **(A1–D1)** and **(H1–H7)** are higher magnification views of the areas boxed in **(A–D)** and **(H)** respectively. Arrowheads in **(D)** and **(H)** delimit the ventral border of habenulae. Thin arrows in **(B)**, **(C-C1)**, **(F)** and **(G)** point towards ventricular cells harboring a pseudo-stratified neuroepithelium organization. Values in the bottom left corner of **(E–F)** refer section numbers from dorsal to ventral habenula levels in **(A–C)** and **(E–G)** and from anterior to posterior habenula levels in **(D,H)**. The levels of horizontal sections are also shown by red lines in the scheme in the upper right panel. Abbreviations: FR, fasciculus retroflexus; HC, habenular commissure; L/R, Left/Right; Pi, pineal organ; Ppi, and parapineal organ. Scale bar = 200 µm.

In order to characterize the general organization of Atlantic salmon habenulae, we first analyzed serial horizontal and transverse sections of this epithalamic structure following nuclear staining and IHC using an antibody directed against acetylated tubulin. This analysis, as well as subsequent ones, was conducted on a series of stages spanning smoltification, in order to assess changes possibly related to this major life transition (Figure 1, Supplementary Figure S1). At all stages studied, the left and right habenulae form well individualized bilateral structures, which protrude from the adjacent thalamus at anterior and dorsal levels (Figure 1A,B,E,F; Supplementary Figure S1A,B,E,F,I,J). At posterior and ventral levels, they are delimited by a ridge along the ventricular zone, within a region exhibiting a typical pseudo-stratified neuroepithelium organization (Figure 1C,C1,D,G,H; Supplementary Figure S1C,D,G,H,K,L). Analyses of the organization of neuronal cell populations highlight differences maintained between ventral and dorsal habenular levels through smoltification, as well as in individuals from the same cohort unable to smoltify (parr lower mode). Relatively small round-shaped cells are observed on both sides at ventro-medial levels (Figure 1C1,H3,H6). At ventro-lateral and dorsal levels, cells appear generally larger and less densely packed (Figure 1B1,H1,H4,H5,H7), except at the level of a dorso-medial, ring-shaped nucleus, lining a well-defined neuropil zone, only visible on the left (Figure 1D1,H2). In line with this observation, the number of nuclei in a given surface area (3700 μm^2^) of transverse sections is statistically higher in ventral territories than in dorsal ones, on the left side as well as on the right side, as assessed by analysis of three parr upper mode and three smolt specimens (*p*-values<5E-02; Supplementary Table S3; Supplementary Figure S1L). The arrangement of neuropil and cellular regions is also generally more complex at dorsal and ventro-lateral levels of the habenulae than at the ventro-medial ones (Figure 1D,H). Altogether, this cellular organization supports a partitioning of habenulae into a ventral domain and a dorsal one, as reported in other teleosts (Amo et al., 2010; Villalón et al., 2012).

### 3.2 Bilateral expressions of *Sskctd12b*, *Sssox1b,* and *Sskiss1* define three largely complementary habenular subdomains

**FIGURE 2:**
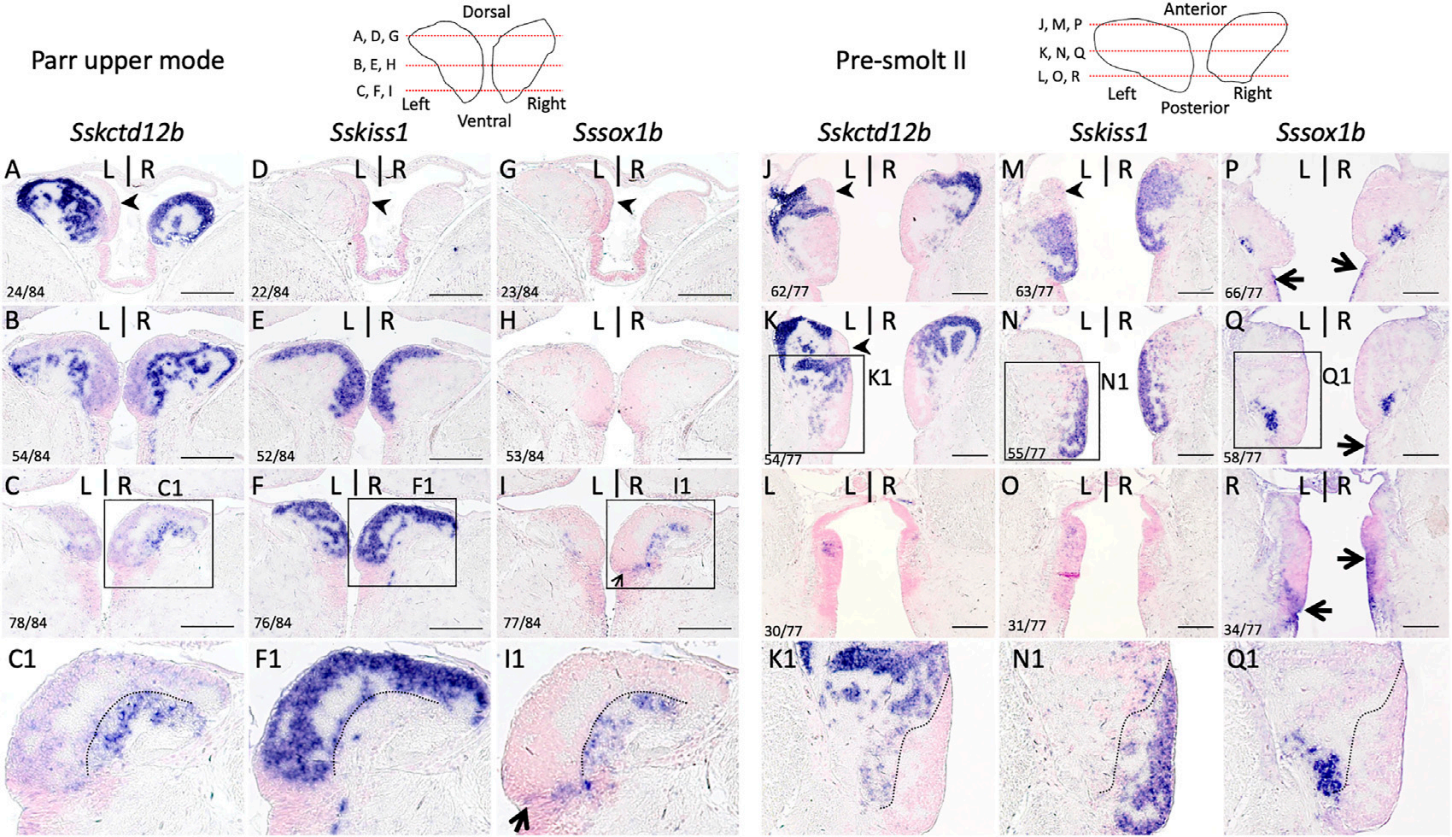
Subdomain organization of habenulae in Atlantic salmon during smoltification. **(A–R)** respectively show sections of parr upper mode and pre-smolt II specimens following ISH with probes for Sskctd12b **(A–C,J–L)**, Sskiss1 **(D–F,M–O)** and SsSox1b **(G–I,P–R)**. **(A–I)** are horizontal sections, **(J–R)** transverse sections. Schemes in the upper line show the levels of the sections along the dorso-ventral **(A–I)** or antero-posterior **(J–Q)** axis as indicated. The numbers of the sections shown along these axes are also indicated in the bottom left corner of the photographs. **(C1,F1,I1)** and **(K1,N1,Q1)** show higher magnifications of the areas boxed in **(C,F,I)** and **(K,N,Q)**. Dotted lines in **(F1)** and **(N1)** and in adjacent sections** (C1,I1)** and **(K1,Q1)** delineate the posterior **(F1)** and dorsal **(N1)** limits of Sskiss1 territory, which largely exclude Sskctd12b and Sssox1b signals. Black arrowheads in **(A,J,K)** point towards a left dorsal territory negative for SsKctd12b and expressing pax6 (see below). Scale bars = 200 µm.

**FIGURE 3:**
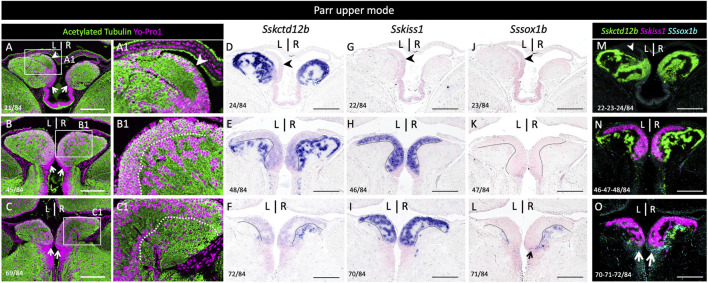
Distinct cellular organizations in Sskctd12b, Sskiss1, Sssox1b habenular territories. **(A–L)** show horizontal sections of habenulae in a parr upper mode following IHC with an antibody directed against acetylated tubulin (green) and Yo-Pro1 staining (purple)** (A–C)**, ISH with probes for Sskctd12b **(D–F)**, Sskiss1 **(G–I)**, Sssox1b **(J–L)**. **(A–L)** are adjacent sections at respectively anterior, medial and posterior organ levels, approximately corresponding to those shown in Figures 2A–C. **(A1)**, **(B1)**, **(C1)** show higher magnifications of the areas boxed in **(A)**, **(B)**, **(C)** respectively. Numbers in the bottom left corner of each panel indicate section numbers along the dorso-ventral axis. A dotted line delimits the Sskiss1 posterior border in **(B,C,E,F,H,I,K,L)**. White **(A)** or black **(D,G,J)** arrowheads point towards the dorsal kctd12b negative territory. Thin arrows point towards Sssox1b positive neural progenitors. **(M,N,O)** show alignments of Sskctd12b, Sskiss1 and Sssox1b territories based on adjacent sections. Same abbreviations as in Figure 1. Scale bars = 200 μm.

**FIGURE 4:**
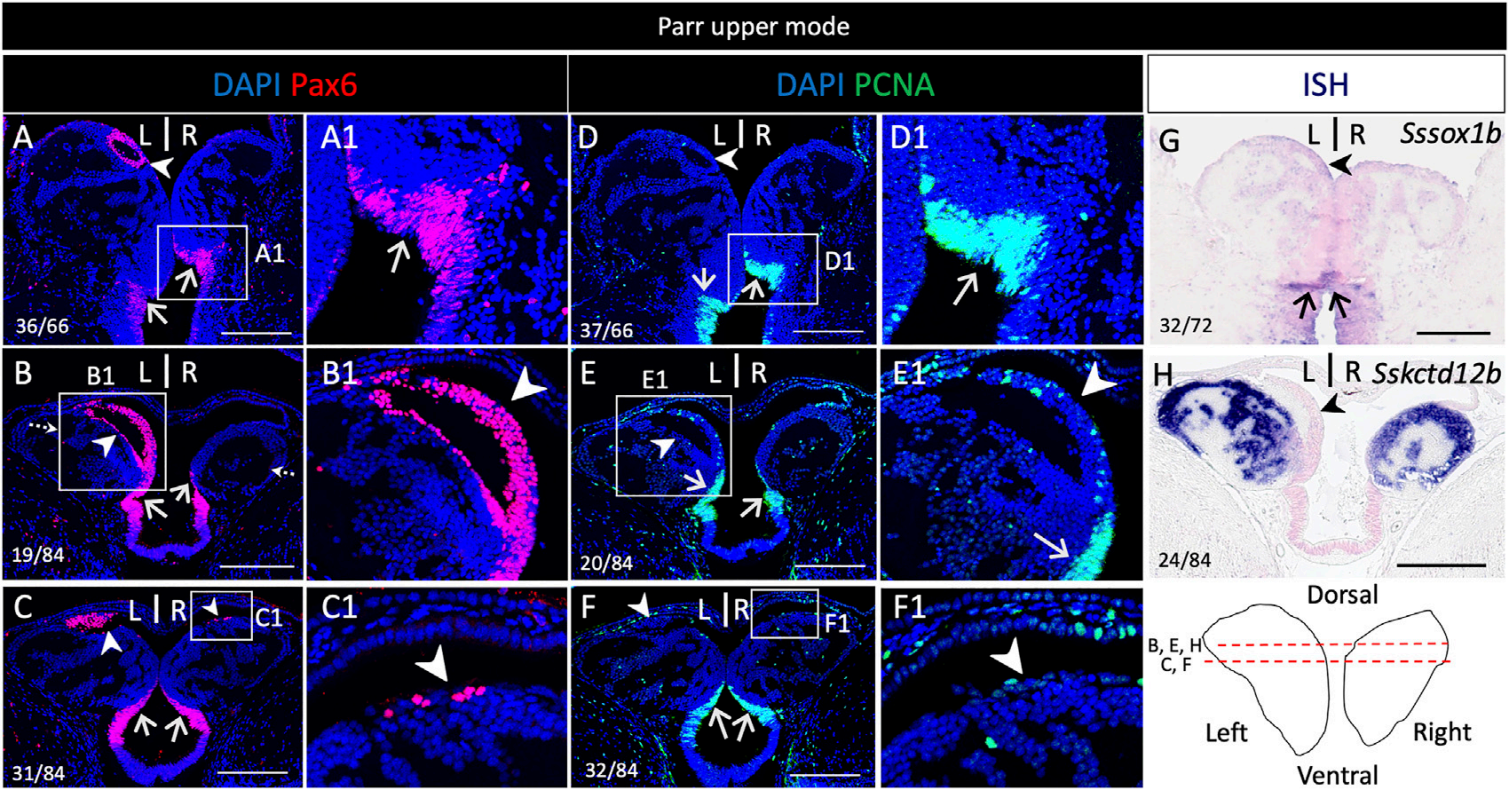
Pax6 expression in neural progenitors and a dorsal neuronal territory in the habenulae of Atlantic salmon. **(A–H)** show sections of a parr upper mode following IHC using antibodies directed against pax6 **(A–C)**, PCNA **(D–F)** and following ISH using probes for Sssox1b **(G)** and Sskctd12b **(H)**. **(A,D,G)** are transverse sections at the level indicated by dotted arrows in **(B)**, **(B–C,E,F,H**) are horizontal sections at the level indicated by red lines on the front view of habenulae schematized in the bottom right panel. **(A1–F1)** are higher magnification views of the territories boxed in **(A–F)**. Arrowheads point towards the left-restricted dorsal nucleus and a smaller, right-sided, dorsal population of clustered cells positive for pax6 but negative for PCNA. Thin arrows point towards posterior and ventral neural progenitors. Same abbreviations as in Figure 1. Scale bars = 200 μm.

In the zebrafish, *kiss1* is broadly expressed in the ventral habenula, while *sox1a* and *sox1b* show more discrete ventral territories, with an additional highly specific parapineal *sox1a* signal (Kitahashi et al., 2009; Servili et al., 2011; Pandey et al., 2018; Lekk et al., 2019). While the paralogous genes *ron*, *lov* and dex are expressed in various dorsal habenula territories in the zebrafish, no *kctd12b* expression has been reported in this species, but this gene shows a highly specific medial habenula territory in the mouse as well as in a cartilaginous fish, suggesting an ancient origin for this territory in gnathostomes (Gamse et al., 2005; Aizawa et al., 2007; Metz et al., 2011; Lagadec et al., 2015). In order to more accurately describe habenula organization in Atlantic salmon, we conducted ISH on sections using probes for Atlantic salmon orthologues of zebrafish *kctd12b, kiss1*, *sox1a* and *sox1b* (Supplementary Table S2; phylogenies in Supplementary Figure S2). Very similar results were obtained at all smoltification stages (Figure 2; Supplementary Figure S3). *Sskctd12b* expression spans a broad dorsal territory, excluding the left restricted dorsal nucleus identified by its higher cell density (Figure 2A–C,J,K; Supplementary Figure S3A1,A2,B1,B2,C1,C2,D1,D2), and extends ventrally to lateral habenular levels, albeit with a lower signal intensity (Figure 2B,C,K,L). *Sskiss1* expression is excluded from dorsal territories (Figure 2D,M,N) and occupies a broad ventral territory excluding lateral *Sskctd12b* positive regions with a sharp boundary (Figure 2E,F; compare Figure 2C1,F1,K1,N1; Supplementary Figure S3E1,E2,F1,F2,G1,G2,H1,H2). No *Sssox1a* is observed in the habenulae but *Sssox1b* is expressed in a small cell population, located in ventral-most habenular regions, and laterally to *Sskiss1* territory (Figure 2G–I,P–R; compare Figure 2F1,I1,N1,Q1; Supplementary Figure S3E1,E2,F1,F2,G1,G2,H1,H2,L1,L2). The labeled territory is distinct from the *Sskctd12b* expression observed at this level, even though some overlap cannot be excluded (compare Figure 2C1,I1,K1,Q1). In order to test whether the partitioning of habenulae in *Sskctd12b*, *Sskiss1* and *Sssox1b* territories correlates with the differences observed in cytoarchitecture between ventral and dorsal domains, we compared cell organization between adjacent sections submitted either to ISH for these markers, or to Yo-Pro 1 staining/acetylated tubulin IHC labeling (Figure 3). The *Sskctd12b* territory correlates with the population of large, loosely packed cells, with the left restricted dorsal nucleus negative for this gene exhibiting a more dense cell population (compare Figure 3A,A1,B,B1,D,E). In ventral habenular regions, the *Sskiss1* territory is superimposable with medial regions characterized by small and tightly packed cells (compare Figure 3B,B1,C, C1,H,I) and excludes dispersed cells positive for *Sskctd12b* and *Sssox1b* at ventral-most levels (Figure 3C,C1,F,I,L). Alignment of adjacent sections confirm that *Sskctd12b* and *Sskiss1* expressions define broad, adjacent territories, excluding each other and respectively prevailing at dorsal and ventral levels, and that *Sssox1b* is expressed in a minor distinct and ventral cell population (Figure 3M–O; Supplementary Figure S4). We refer hereafter to the *Sskctd12b* domain plus left restricted nucleus as dHb, and to the *Sskiss1* plus *Sssox1b* domains as vHb. Additional signals outside the habenulae include *Sssox1a* and *Sssox1b* expressions in the parapineal, but not the pineal organ, persisting throughout smoltification (Supplementary Figure S5A–G,A1–G1). A relatively faint *Sssox1b* signal is also consistently present in the ventricular pseudostratified neuroepithelium observed posteriorly, at all stages analyzed (Supplementary Figure S4H–L,F,G; see also Figure 4G below).

### 3.3 Pax6 expression includes the dorsal left restricted nucleus in addition to neural progenitors

In order to unambiguously identify neural progenitors, we next analyzed PCNA (Proliferating cell nuclear antigen) and pax6 distribution by IHC on habenula sections (Figure 4 and Supplementary Figure S6). At all stages analyzed (lower and upper mode, smoltifying fish), PCNA and pax6 are co-expressed in the posterior and ventral cell population exhibiting a pseudo-stratified neuroepithelial-like organization described above, in line with a neural progenitor identity (Figure 4A–F,A1,D1; Supplementary Figure S6A1,B1,A2-H2, S7). This suggests that during smoltification, habenulae primarily grow from this zone, referred to hereafter as posterior growth zone (PGZ). An additional major pax6 territory is observed in the left dorsal ring of cells negative for *SsKctd12b* (Figure 4G)*,* distinguished dorsally by its relatively high cell density (Figure 4A,B,B1; Supplementary Figure S6A1,A2,C1,E1,E2,G1,G2). This nucleus is PCNA negative (compare Figure 4A–F; compare Supplementary Figure S6A1–H2). While this characteristic nucleus could not be detected based on morphology on the right, a small number of clustered cells, similarly positive for pax6 but negative for PCNA, are present in the right habenula, at the same dorsal position (Figure 4C,C1,F,F1; Supplementary Figure S6A2–H2,A2′–H2′).

### 3.4 Quantitative analyses of asymmetries

**FIGURE 5:**
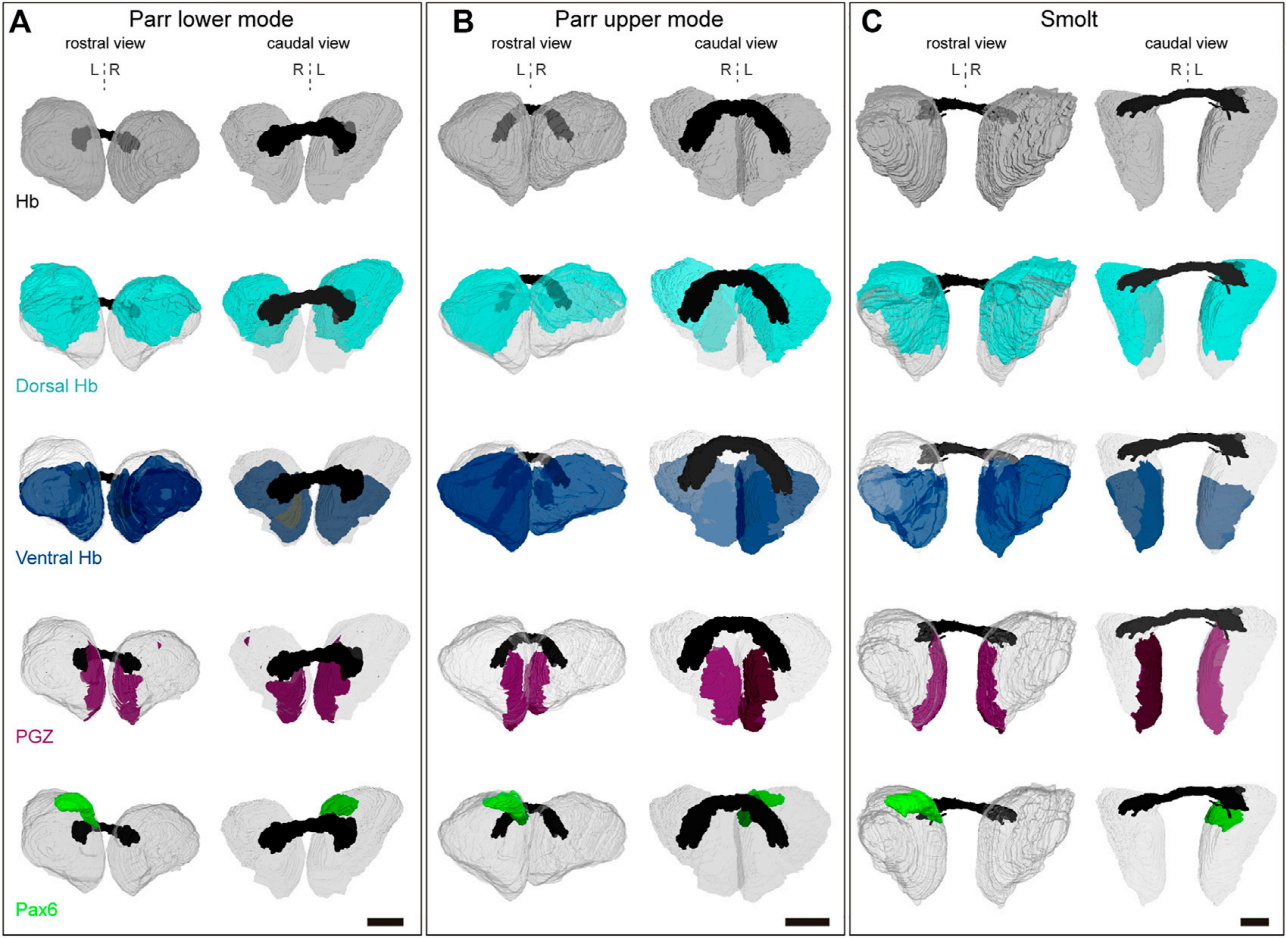
3D reconstructions of habenulae. **(A–C)** show 3D reconstructions of habenulae respectively in a parr lower mode, parr upper mode and smolt, with rostral and caudal views on the left and on the right as indicated. The whole habenulae are shown in grey (first line), the dorsal habenulae (including the pax6 dorsal left nucleus) in light blue (second line), the ventral habenulae (comprising kiss1 and sox1b positive territories) in dark blue (third line), the posterior growth zone (PGZ) in purple (fourth line) and the pax6 left dorsal nucleus in green (fifth line). The habenular commissure is in black. A dotted vertical line indicates the midline. Abbreviations: Hb, habenulae; L, left; R, right. Scale bars = 150 μm.

We next used 3D reconstructions to quantify volumetric asymmetries of Atlantic salmon habenulae and of their sub-territories. To do so, successive sections submitted to either ISH with *Sskctd12b*/*Sskiss1*/*Sssox1b* probes, or to IHC with antibodies directed against pax6 and nuclear staining were aligned (Figure 5). Volumes were quantified for the following territories: left and right habenulae, dorsal habenula (including pax6 territory or not), ventral habenula (*Sskiss1* plus *Sssox1b* territories excluding the PGZ), the PGZ, the pax6 positive left dorsal nucleus and nuclei. This analysis was conducted in one specimen prior to, at the onset and at the end of smoltification (parr lower mode, parr upper mode and smolt, respectively; Table 1 and Table 2). Despite substantial changes in habenula volumes between these three specimens (with a factor exceeding 2.5 between the parr upper mode and smolt analyzed), the relative volumes occupied by each territory within the left or right habenulae are similar in the three specimens, with larger dorsal than ventral habenulae (respectively 50%–60% *versus* 31%–46% of ipsilateral habenula) in all specimens and on both sides (Table 1). The relative volume of either the PGZ or the left sided pax6 nucleus do not significantly change between the specimens (3%–4%) (Table 1). The only notable difference may be a substantially lower relative volume of nuclei in the left habenula of smolt (46%) *versus* parrs (57% and 55% for lower and upper mode parrs respectively) (Table 1), but analyses of a higher number of samples to assess inter-individual differences are required for definite conclusions in this respect. Concerning asymmetries, their degree, as assessed by laterality indexes, and is very similar for all specimens for several territories (Table 2). The left habenula is larger than the right one in all specimens tested, with similar degrees of asymmetry between all specimens analyzed, as revealed by laterality indexes (−9.5 < LI_Hb_ < −10.4; Table 2). The dorsal habenula (including the pax6 left nucleus) mainly accounts for this difference between the left and right habenulae (−15.8 < LI_dHb_ < −11.6; Table 2), asymmetries in vHb appearing less marked, albeit always with the same directionality (−5.8 < LI_vHb_ < −0.7; Table 2). A relatively high size difference is maintained between dorsal, *kctd12b* expressing, left and right domains, even without taking the pax6 left restricted nucleus into account (−12.7<LI_dHb-pax6_<−8.6; Table 2). Finally, some differences in laterality indexes vary between the three specimens, as observed for PGZ or nuclei volumes, which exhibit very low degrees of asymmetry in the smolt (LI_PGZ_ = −2.8 and LI_Nuclei_ = −0.3) compared to the parr upper and lower modes (LI_PGZ_ = −13.8/−10.0 and LI_Nuclei_ = −8.7/−12.2; Table 2). Whether this observation reflects a loss of PGZ and nuclei volumes during smoltification remains to be assessed by analyzing additional specimens.

**TABLE 1:** Quantification of habenula subdomain volumes in Atlantic salmon. Volumes were quantified using habenula 3D reconstructions for one parr lower mode, one par upper mode and one smolt as indicated. They are indicated for the following subdomains of the left and right habenulae (Hb) (columns 2–3 and 4–5 respectively): dorsal territory including the pax6 left nucleus (i.e., *Sskctd12b* positive territory plus pax6 nucleus: Dorsal Hb + pax6), or excluding the pax6 left nucleus (i.e., *Sskctd12b* positive territory only: Dorsal Hb-pax6), ventral territory (i.e., *Sssox1b* plus *Ssktd12b* positive territories: Ventral Hb), PGZ (posterior growth zone), nuclei (selected based on Yo-Pro1 staining) and pax6 (pax6 left nucleus). The ratio occupied by each domain within the left (column 3) or right habenula (column 5) is indicated as percentage (%). Volumes are expressed in μm^3^*10^6^.

	Left	%	Right	%	
Hb	36.5		29.6		Parr lower mode
Dorsal Hb (+pax6)	21.1	58	15.4	52	
Dorsal Hb (-pax6)	19.8	54	15.4	52	
Ventral Hb	11.3	31	11.1	38	
PGZ	1.2	3	0.9	3	
Nuclei	20.7	57	17.4	59	
Pax6	1.3	4			
Hb	26.9		22.2		Parr upper mode
Dorsal Hb (+pax6)	14.7	55	11.2	50	
Dorsal Hb (-pax6)	13.8	51	11.2	50	
Ventral Hb	12.2	46	10.9	49	
PGZ	0.9	3	0.7	3	
Nuclei	14.9	55	11.7	53	
Pax6	0.9	3			
Hb	72.6		59.4		Smolt
Dorsal Hb (+pax6)	43.3	60	34.3	58	
Dorsal Hb (-pax6)	40.7	56	34.3	58	
Ventral Hb	26.9	37	24.8	42	
PGZ	2.1	4	2.0	3	
Nuclei	33.6	46	33.3	56	
Pax6	2.6	4			

**TABLE 2:** Quantification of habenula subdomain volumes in Atlantic salmon. Volumes were quantified using habenula 3D reconstructions for one parr lower mode, one par upper mode and one smolt, same specimen as in Table 1. Abbreviations for subdomains are the same as in Table 1. Volumes for left subdomains, right subdomains and corresponding left plus right subdomains are indicated in columns 2, 4 and 6 respectively. The ratio occupied by the left or right component relative to their sum is indicated as percentage (%) in columns 3 and 5, the corresponding laterality index (LI, calculated as LI = x100 [Right domain volume-Left domain volume]/[Right + Left domain volumes] is shown in column 7. Volumes are expressed in μm^3^*10^6^.

	Left	%	Right	%	Total	Li	
Hb	36.5	55	29.6	45	66.1	−10.4	Parr lower mode
Dorsal Hb (+ pax6)	21.1	58	15.4	42	36.5	−15.8	
Dorsal Hb (− pax6)	19.8	56	15.4	43	35.1	−12.6	
Ventral Hb	11.3	50	11.1	50	22.4	−0.7	
PGZ	1.2	57	0.9	43	2.0	−13.8	
Nuclei	20.7	54	17.4	46	38.0	−8.7	
Pax6	1.3	100				−100	
Hb	26.9	55	22.2	45	49.1	−9.5	Parr upper mode
Dorsal Hb (+ pax6)	14.7	57	11.2	43	25.9	−13.7	
Dorsal Hb (− pax6)	13.8	55	11.2	45	24.9	−10.5	
Ventral Hb	12.2	53	10.9	47	23.0	−5.8	
PGZ	0.9	55	0.7	45	1.6	−10.0	
Nuclei	14.9	56	11.7	44	26.6	−12.2	
Pax6	0.9	100				−100	
Hb	72.6	55	59.4	45	132.0	−10.0	Smolt
Dorsal Hb (+ pax6)	43.3	56	34.3	44	77.7	−11.6	
Dorsal Hb (− pax6)	40.7	54	34.3	46	75.1	−8.6	
Ventral Hb	26.9	52	24.8	48	51.7	−4.1	
PGZ	2.1	51	2.0	49	4.1	−2.8	
Nuclei	33.6	50	33.3	50	66.9	−0.3	
Pax6	2.6	100				−100	

### 3.5 A pax6 positive habenular territory is widespread across teleosts but undetectable in the spotted gar and the reedfish

**FIGURE 6:**
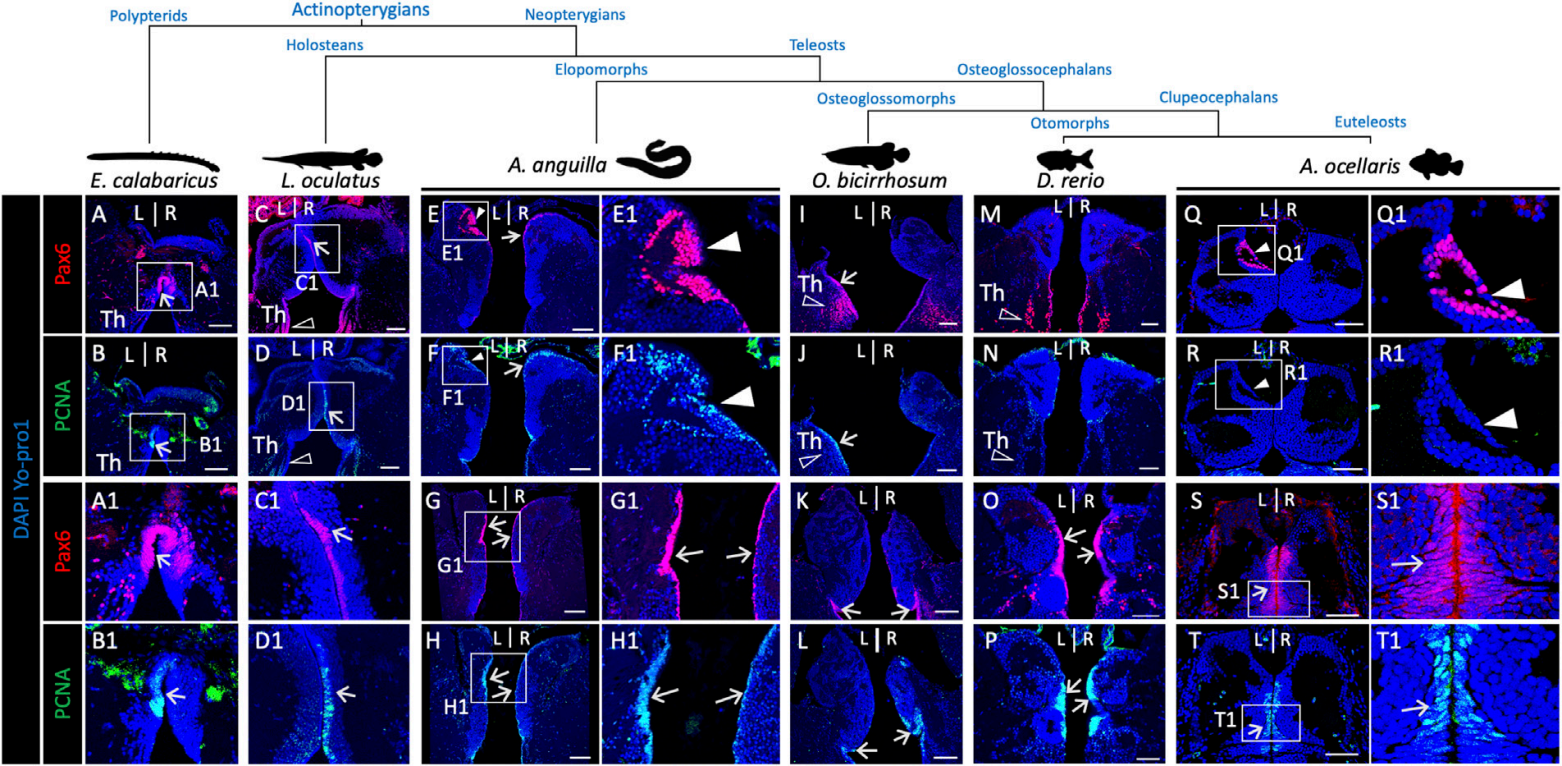
Pattern of presence/absence of an asymmetric pax6 dorsal nucleus in actinopterygians.** (A-R)** Transverse sections of habenulae in the reedfish E. calabaricus [**(A,B)**; 20 cm juvenile], spotted gar L. oculatus [**(C,D)**; 8.5 cm juvenile], European eel A. anguilla [**(E–H)**; yellow resident stage], silver arowana O. bicirrhosum [**(I–L)**; 7 cm juvenile], zebrafish D. rerio [**(M,P)**; adult] and false clownfish A. ocellaris [**(Q–T)**; stage 6], following IHC with antibodies directed against pax6 **(A,C,E,I,M,Q,G,K,O,S)** and PCNA **(B,D,F,J,N,R,H,L,P,T)**. **(A1–H1)**, **(Q1–T1)** are higher magnification views of the territories boxed in **(A–H)**, **(Q–T)**. Arrows point towards pax6 and PCNA positive neural progenitors, white arrowheads point to left restricted pax6 positive and PCNA negative dorsal nuclei observed in the European eel and false clownfish as in the Atlantic salmon. Opened arrowheads indicate thalamic Pax6 positive neuronal populations. Scale bars = 100 μm in **(A–L)** and 50 μm in **(M–T)**.

The presence of an asymmetric pax6 positive habenula nucleus, as observed in the Atlantic salmon, has not been reported thus far in the zebrafish. In order to address the phylogenetic distribution of this trait, we analyzed pax6 expression by IHC on habenula sections from a broad sampling of teleosts comprising an elopomorph, the European eel (*Anguilla*), an osteoglossomorph, the silver arowana (*Osteoglossum bicirrhosum*), an otomorph, the zebrafish and another euteleost than the Atlantic salmon, the false clownfish (*Amphiprion ocellaris*). Two actinopterygians, which do not belong to teleosts, the reedfish *Erpetoichthys calabaricus* and the spotted gar *Lepisosteus oculatus,* were also analyzed in order to address the origin of this trait in ray-finned fishes (Figure 6). In all species tested, pax6 is expressed in thalamic or habenular ventricular cells, also positive for PCNA, in line with a neural progenitor identity (Figure 6A–D,G–H,K,L,O,P,S,T; Supplementary Figure S8C2,C3,G2,G3), as well as in other thalamic cell populations (Figure 6C,I,M; Supplementary Figure S8A2,A3,E2,E3,G2,G3). In addition, a dorsal, left restricted ring of pax6 positive cells reminiscent of the territory observed in Atlantic salmon is present in two teleosts, an elopomorph (European eel) and an euteleost (false clownfish) (Figure 6E,E1,Q,Q1). As in Atlantic salmon, no PCNA expression is detected at this level on adjacent sections (Figure 6F,F1,R,R1), while strong PCNA signals are observed at the level of progenitors (Figure 6G,H,S,T). This characteristic pax6 territory remains undetectable in the zebrafish (Figure 6M–P), arowana (Figure 6I–L), spotted gar or reedfish (Figure 6A–D; Supplementary Figure S8).

### 3.6 The partitioning of habenulae is similar between the european Anguilla and Atlantic salmon

**FIGURE 7:**
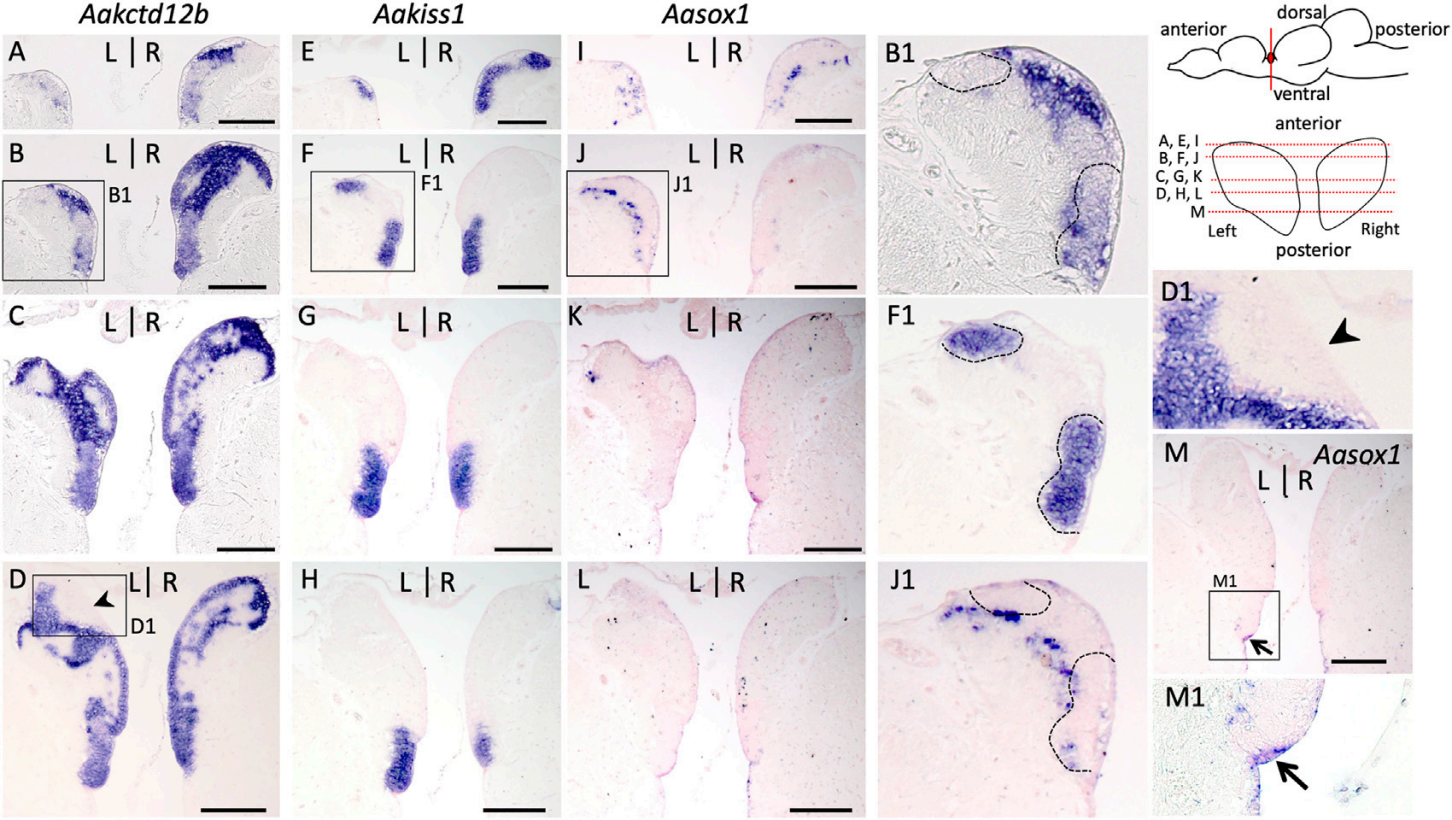
Subdomain organization of habenulae in the European eel. **(A–M)** transverse sections of habenulae in A. anguilla (silver specimens) following ISH with probes for Aakctd12b **(A–D)**, Aakiss1 **(E–H)** and Aasox1 **(I–M)**. The orientation of section planes is shown in a schematic lateral view of A. anguilla brain and section levels along the antero-posterior axis are shown as red dotted lines on a front view of habenulae schematized in the top right corner. **(B1,F1,J1,D1,M1)** are higher magnification views of the areas boxed in **(B,F,J,D,M)**. Black arrowheads point towards the left dorsal nucleus positive for pax6, and negative for PCNA and Aakctd12b. The thin arrow in **(M,M1)** points towards Aasox1 positive neural progenitors. The dotted line in **(F1)** and adjacent sections **(B1)** and **(J1)** delimits the Aakiss1 positive territory, which is adjacent to, but largely excludes Aasox1 expression at anterior-most levels of the habenulae. At this level, Aakiss1 expands dorsally but it is restricted to the organ ventral parts at medial to posterior levels. Scale bars = 200 μm.

In order to further assess the conservation of the relative location of the pax6 dorsal territory within the left habenula, we analyzed the subdomain habenular organization in the European eel, which by its phylogenetic position provides a comparative reference to identify ancestral traits of teleosts. Analysis was conducted using IHC with an antibody directed against acetylated tubulin and ISH analyses with probes for *Aakctd12b*, *Aakiss1* and, *Aasox1* (the only *sox1* form retained in this species, whose relationship with the zebrafish *sox1a* and *sox1b* paralogues could not be resolved: see phylogeny and synteny analysis in Supplementary Figure S9). This analysis shows that in this species as in the Atlantic salmon, the cellular architecture varies between dorsal and ventral habenular regions, cells being larger, and more loosely packed in dorsal than in ventral regions except at the level of the pax6 dorsal nucleus (Supplementary Figure S10A–C; compare Supplementary Figure S10B1–B4). A ventricular cell population co-expressing pax6 and PCNA also persists at this stage (Supplementary Figure S10E–F2). ISH analysis shows that *Aakctd12b* is broadly expressed in the habenulae, albeit with a higher signal intensity in dorsal regions, characterized by a lower cell density (Figures 7A–D). However, it completely excludes the dorsal territory expressing pax6 (Figure 7D1). In contrast, *Aakiss1* expression is restricted to a ventral territory, delimited by a sharp dorsal boundary, at most organ levels (Figure 7G,H) except at anterior-most ones, where the gene is broadly expressed (Figure 7E,F,F1). *Aasox1* transcripts are only observed in anterior habenula regions (compare Figure 7I–L), in dispersed cells, lateral to *Aakiss1* expression domain as in Atlantic salmon (Figure 7E,F,I,J). Comparisons of labeled territories between adjacent sections show that at anterior-most habenula levels, *Aakiss1* territory largely excludes the domains exhibiting *Aasox1* and higher intensity *Aakctd12b* signals (Figure 7B1,F1,J1). Altogether, this analysis highlights very similar subdomain organizations of the habenulae between the Atlantic salmon and European eel. Of note *Aasox1* expression also includes a minor ventral and posterior population of ventricular cells, positive for pax6 and PCNA in line with a neural progenitor identity and extending in thalamic regions (Figure 7M,M1; Supplementary Figure S10D,D2), but no signal can be detected in the parapineal organ, located to the left of the midline as in Atlantic salmon or zebrafish (Supplementary Figure S10D,D1).

## 4 Discussion

Habenular asymmetries in teleosts have thus far been essentially viewed through the prism of the zebrafish. The description of habenular cytoarchitecture, subdomain organization and asymmetries in the Atlantic salmon highlight conservations but also major differences with this reference organism which provides a renewed view of habenular asymmetry evolution in teleosts.

A partitioning of habenulae into dorsal and ventral components as described in the zebrafish has been reported in several members of euteleosts, based on subtle differences in cytoarchitecture (Amo et al., 2010; Villalón et al., 2012). Our analysis in Atlantic salmon confirms the conservation of this subdivision on this criterion, ventral habenula territories containing smaller and more densely packed cells than dorsal ones, exactly as observed in the zebrafish or the redtail sharkminnow *Epalzeorhynchos bicolor* (Villalón et al., 2012). Similarly, habenular asymmetries are primarily confined to dorsal territories as in the zebrafish, suggesting that an escape of vHb cells from signals secreted by the parapineal may take place in both species (Beretta et al., 2013). This partitioning is supported by the identification of conserved subdomain signature markers. A dorsal habenular expression of members of the *kctd8/12a/12b/16* family, is shared by the Atlantic salmon and European eel, as shown by *kctd12b* expression, in line with a conservation in teleosts. This is not unexpected as the same conclusion applies to mammalian medial habenulae, viewed as the homologue of the zebrafish dorsal habenula (Amo et al., 2010; Metz et al., 2011; Hashikawa et al., 2020). The Atlantic salmon and European eel also exhibit similar relative organizations of ventral territories, with a major, highly specific *kiss1* positive territory but also a more minor *sox1a/b* expressing cell population at lateral organ levels. While an habenular *kiss1* expression has never been detected in tetrapods to our knowledge, this organization may be largely conserved across teleosts. *Kiss1*, a major and well documented ventral zebrafish habenula marker in the zebrafish, but also *sox1a/b*, have indeed been identified as signature markers of distinct ventral cell clusters, in an scRNA-seq habenula characterization conducted in this species (Pandey et al., 2018). Altogether, these data suggest that the partitioning of habenulae into a dorsal *kctd* expression territory, and a ventral one, comprising *kiss1* and *sox1* positive cell populations, is an ancient and conserved feature of teleosts.

This overall conservation contrasts with the relatively complex pattern of presence/absence of the additional, almost completely left restricted, pax6 positive nucleus, detected in two euteleosts (Atlantic salmon and false clownfish) and an elopomorph (European eel), but undetectable in an otomorph (zebrafish), or in an osteoglossomorph (silver arowana). In view of the absence of this characteristic cell population in the two actinopterygians analyzed outside teleosts (reedfish and spotted gar), this trait distribution may reflect an ancient innovation of teleosts, lost several times during their evolution. However, it is difficult to exclude recurrent independent rises in different lineages of teleosts, possibly related to combinations of developmental and environmental constraints. Whatever the evolutionary scenario underlying its rise, the detection of this trait in several major lineages of teleosts opens novel questions on asymmetry formation and evolution in the taxon. First, concerning the conservation of habenular asymmetries within teleosts, whether the presence of this additional asymmetry may change the cellular environment in the dorsal habenula with an impact on other asymmetries remains an open question. Our volumetric analyses highlight an asymmetry between Atlantic salmon left and right *kctd12b* territory, with the same size laterality in all three stages analyzed. It will be of interest to test whether a partitioning of this territory into medial and lateral components exhibiting different relative proportions and characterized by the same gene signatures as in the zebrafish is maintained across teleosts. Second, the embryological origin and the mechanisms controlling the formation of the pax6 nucleus remain completely unknown in the absence of a related structure in the zebrafish. In the medaka, the parapineal incorporates into the left habenula during development (Ishikawa et al., 2015). The Atlantic salmon left pax6 nucleus precisely corresponds by its characteristic morphology and position to a serotoninergic nucleus expressing opsins and activated by light (Sandbakken et al., 2012; Eilertsen et al., 2021), suggesting similarities to the parapineal organ. However, a parapineal integration similar to the process described in the medaka into the left habenula is unlikely to account for its presence in the Atlantic salmon or European eel, which both harbor distinct parapineal organs. Whether the pax6 nucleus and parapineal could derive from related populations of progenitors remains to be assessed. Concerning the mechanisms underlying the formation of the latter, while a highly specific parapineal *sox1a/b* expression was detected in the Atlantic salmon, we were unable to detect a signal for the only *sox1* paralogue retained in European eel at this level. Even though we cannot exclude a transient expression during development, this questions the conservation of a *sox1*-and parapineal-dependent control of asymmetry formation as in the zebrafish (Concha et al., 2003; Gamse et al., 2003; Regan et al., 2009). Whether Nodal signaling, which has lost ancestral roles in habenular asymmetry formation but regulates parapineal cell numbers in the zebrafish (Garric et al., 2014; Lagadec et al., 2015), may be involved in the formation of this nucleus, remains an intriguing possibility. Finally, concerning the biological significance of this novel asymmetry, the presence of a photosensitive left restricted nucleus as is the case in Atlantic salmon may add complexity to the integration of visual signals, known to primarily take place on the left, in the zebrafish habenula (Dreosti et al., 2014). We note that in our study, the presence of this additional nucleus correlates with species endowed with complex life cycles and light dependent phase transitions. During these transitions, these species go through major morphological, physiological and behavioral changes, regulated by environmental factors including photoperiod and temperature, and involving neural circuits remodeling (Ebbesson and Braithwaite, 2012; Strand et al., 2018; Cresci et al., 2020; Schalm et al., 2021). As a major forebrain center integrating environment and complex behavioral, cognitive and emotional organismal responses, the habenulae may be involved in these modifications. The pax6 nucleus reported here provides an attractive system to address these issues, and its biological significance should be further assessed by expanding the sampling of species analyzed and conducting direct functional analyses in tractable experimental models such as the false clownfish (Roux et al., 2020). The medaka, another tractable system, might also add an extra layer of complexity to the possible function of the asymmetric pax6 domain. In medaka larvae, a small left-sided habenular subnucleus showing a cytoarchitecture and a dorso-medial position strikingly similar to the pax6 habenular domain found in Atlantic salmon, false clownfish and European eel, has been described as the main receptor for parapineal projections (Signore et al., 2009). Although the presence of pax6 in the medaka habenula still needs to be corroborated, this observation opens the possibility that the asymmetric pax6 domain represent a structural specialization of the left habenula that mediates the intraepithalamic integration of parapineal activity in the habenular circuit, which will merit attention in future studies. In conclusion, by revealing a highly evolvable asymmetric trait in teleosts, this analysis highlights the importance of exploiting biological diversity to gain a comprehensive view of habenular asymmetries in their mechanistic and ecological aspects.

## Data Availability

The original contributions presented in the study are included in the article/Supplementary Material, further inquiries can be directed to the corresponding author.
