# Supplementary material for: Diversification of habenular organization and asymmetries in teleosts: Insights from the Atlantic salmon and European eel
Source: Front Cell Dev Biol. 2022 Nov 3;10:1015074. doi: 10.3389/fcell.2022.1015074 (PMC9671474; doi:10.3389/fcell.2022.1015074)
Supplement: Supplementary file 1 [file DataSheet7.PDF]

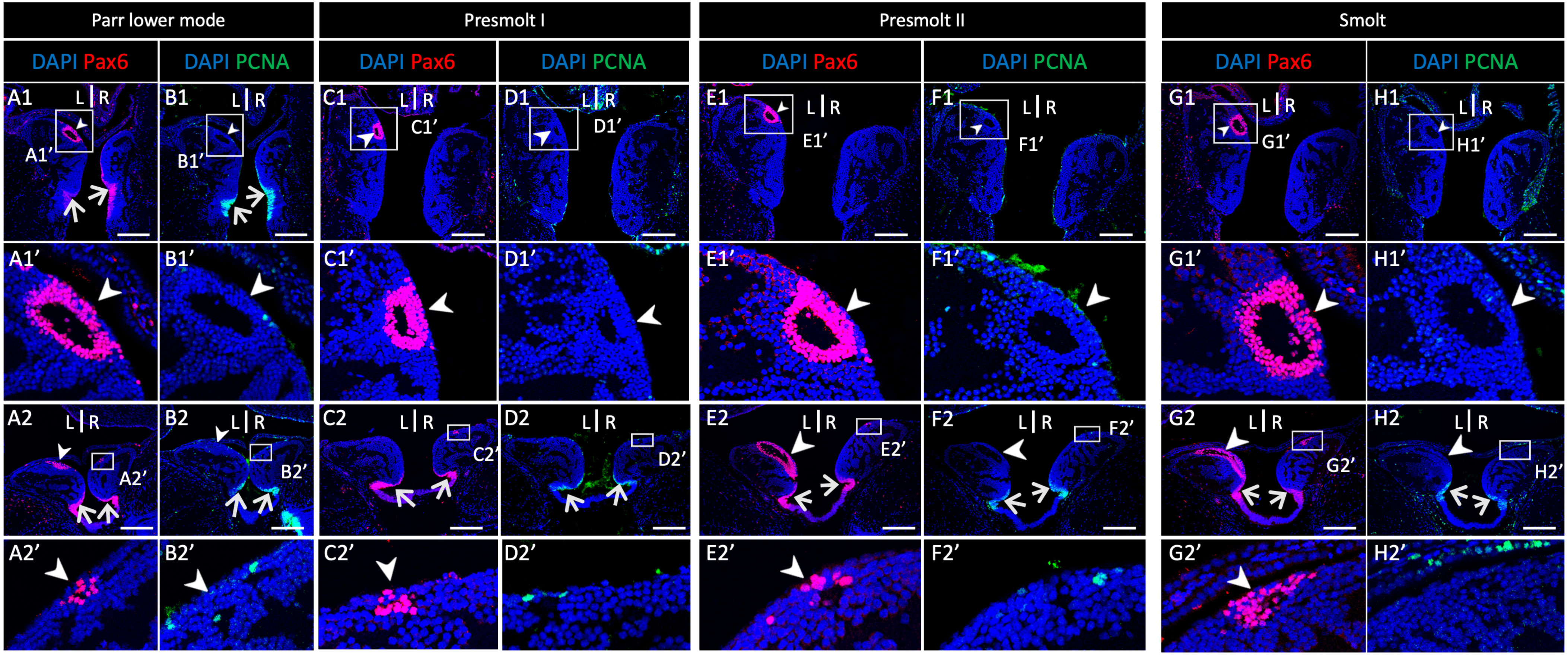

Supplementary Figure 6. Expression of pax6 in neural progenitors and in a left dorsal nucleus. (A-H) show transverse (A1,B1,C1,D1,E1,F1,G1,H1) and horizontal (A2,B2,C2,D2,E2,F2,G2,H2) sections of Parr lower mode (A,B), pre-smolt I (C,D), pre-smolt II (E,F) and smolt (G,H) Atlantic salmon specimens, following IHC with an antibody directed against pax6 (A,C,E,G) and PCNA (B,D,F,H). (A1',B1',C1',D1',E1',F1',G1',H1') and (A2',B2',C2',D2',E2',F2',G2',H2') show higher magnifications of the areas boxed in (A1,B1,C1,D1,E1,F1,G1,H1) and horizontal (A2,B2,C2,D2,E2,F2,G2,H2). Vertical lines indicate the midline. White arrowheads point to pax6 positive neurons negative for PCNA, which form a distinct nucleus in the dorsal left habenula but are also visible in a mirror position on the right. Thin arrows point to neural progenitors. L, left; R, right. Scale bars=200µm.
