# Supplementary material for: Diversification of habenular organization and asymmetries in teleosts: Insights from the Atlantic salmon and European eel
Source: Front Cell Dev Biol. 2022 Nov 3;10:1015074. doi: 10.3389/fcell.2022.1015074 (PMC9671474; doi:10.3389/fcell.2022.1015074)
Supplement: Supplementary file 3 [file DataSheet2.PDF]

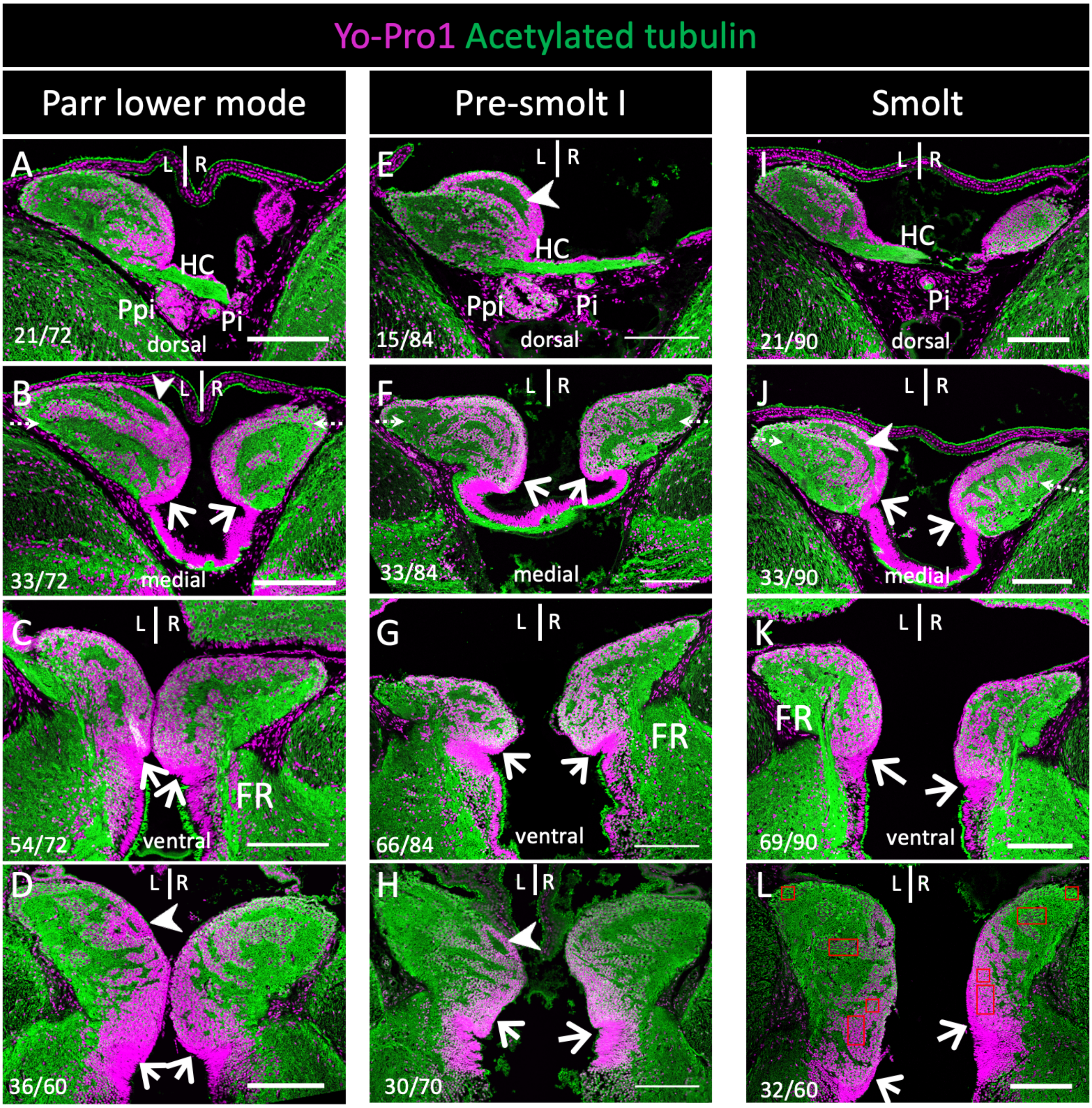

Supplementary Figure 1. Cytoarchitecture of habenulae in Atlantic salmon. (A-D), (E-H) and (I-L) show histological sections of the habenulae in parr lower mode, pre-smolt I and smolt respectively, following nuclear staining using Yo-Pro1 (purple) and IHC with an antibody directed against acetylated tubulin (green). (A-C,E-G, I-K) are horizontal sections at dorsal (A,E,I), median (B,F,J) and ventral (C,G,K) organ levels; (D,H,L) are transverse sections at the level indicated by dotted arrows in (B,F,J). Red boxes in (L) show an example of the areas taken into account for the comparison of nuclei densities between dorsal and ventral territories (see Supplementary Table 3). Arrowheads point towards a dorsal pax6 positive nucleus, restricted to the left and observed at all stages analyzed. Thin arrows point towards ventricular cells harboring a pseudo-stratified neuroepithelium organization. A vertical bar indicates the midline. Values in the bottom left corner refer to section numbers from dorsal to ventral habenula levels in (A-C,E-G, I-K) and from anterior to posterior habenula levels in (D,H,L). Abbreviations: FR, fasciculus retroflexus; HC, habenular commissure; L, left; R, right; Pi, pineal organ; Ppi, parapineal organ. Scale bar=200 µm .
