# Supplementary material for: Diversification of habenular organization and asymmetries in teleosts: Insights from the Atlantic salmon and European eel
Source: Front Cell Dev Biol. 2022 Nov 3;10:1015074. doi: 10.3389/fcell.2022.1015074 (PMC9671474; doi:10.3389/fcell.2022.1015074)
Supplement: Supplementary file 4 [file DataSheet4.PDF]

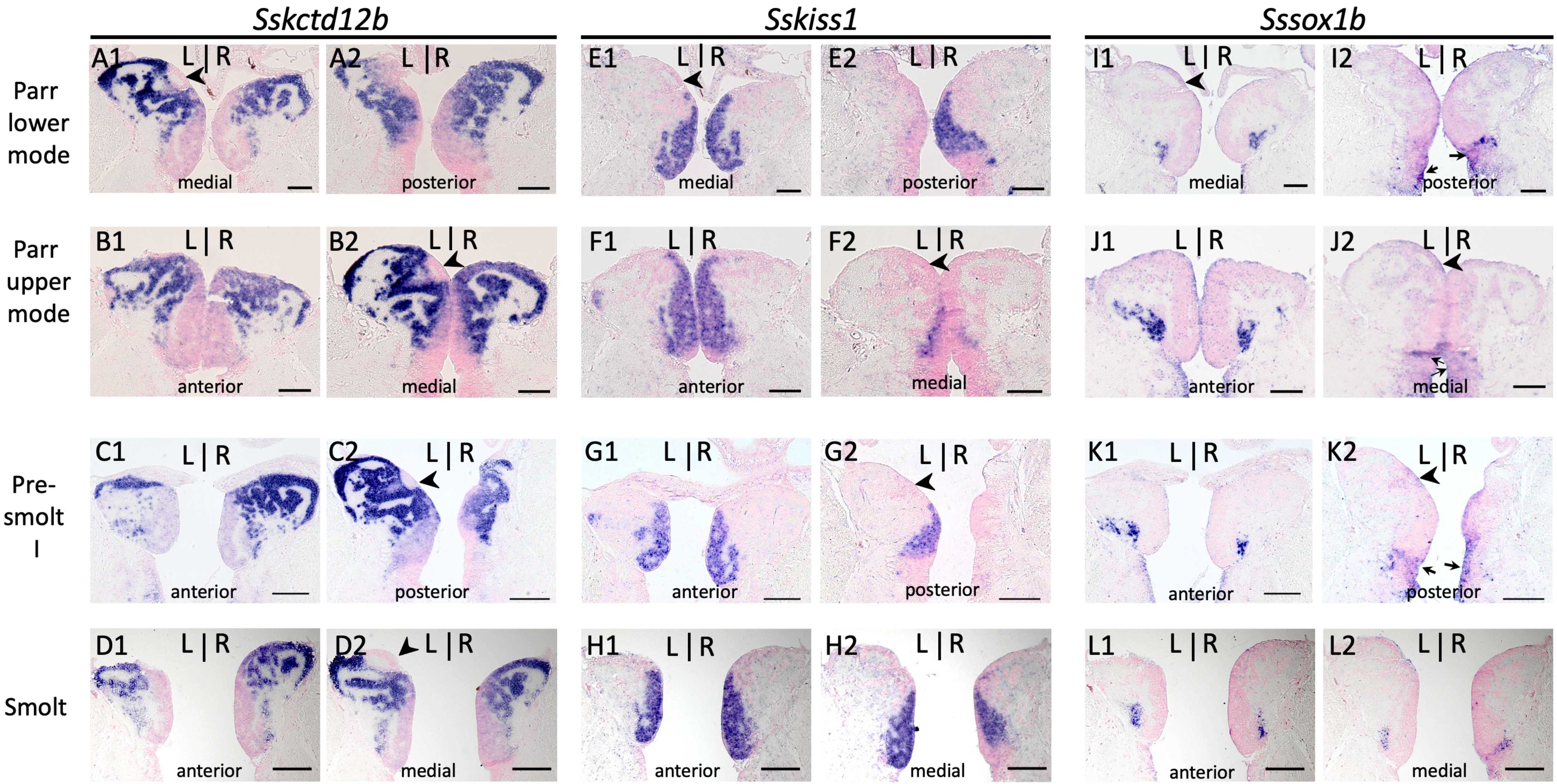

Supplementary Figure 3. Subdomain organization of habenulae in Atlantic salmon during smoltification. (A1-A2,B1-B2,C1-C2,D1-D2), (E1-E2,F1-F2,G1-G2,H1-H2) and (I1-I2,J1-J2,K1-K2,L1-L2) respectively show transverse sections following ISH with probes for *Sskctd12b*, *Sskiss1* and *Sssox1b*. The stages analyzed are the following: (A1,A2,E1,E2,I1,I2), parr lower mode; (B1,B2,F1,F2,J1,J2), parr upper mode; (C1,C2,G1,G2,K1,K2), pre-smolt I; (D1,D2,H1,H2,L1,L2), smolt. The levels of the sections (anterior, medial or posterior) are indicated. A black arrowhead points towards a left dorsal territory negative for *SsKctd12b* and expressing Pax6. Thin arrows indicate a *sox1b* signal in neural progenitors. Scale bars=200µm.
