# Supplementary material for: Diversification of habenular organization and asymmetries in teleosts: Insights from the Atlantic salmon and European eel
Source: Front Cell Dev Biol. 2022 Nov 3;10:1015074. doi: 10.3389/fcell.2022.1015074 (PMC9671474; doi:10.3389/fcell.2022.1015074)
Supplement: Supplementary file 5 [file DataSheet6.PDF]

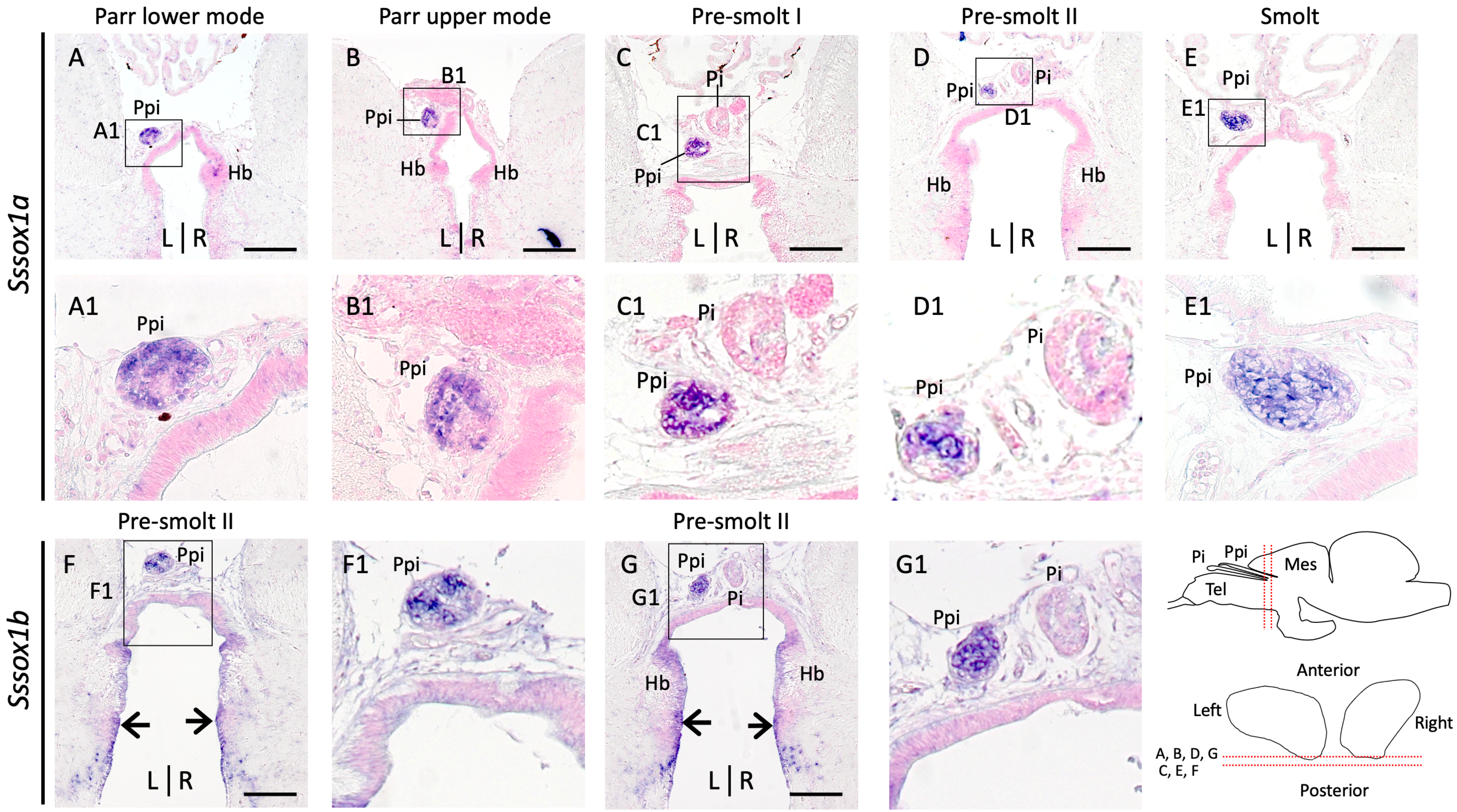

Supplementary Figure 5. Expression of *Sssox1a/b* in the parapineal organ in the Atlantic salmon. (A-G) show transverse sections of Atlantic salmon forebrains posterior to the habenulae following ISH with probes for *Sssox1a* (A-E) and *Sssox1b* (F-G). The stages analyzed are the following: (A), parr lower mode; (B), par upper mode; (C), pre-smolt I; (D,F,G), pre-smolt II; (E), smolt. The levels of the sections are shown as red dotted lines on a lateral view of the brain and a dorsal view of the habenulae schematized in the bottom right panel. Arrows point towards a *Sssox1b* signal in thalamic neural progenitors. A vertical bar indicates the midline. (A1,B1,C1,D1,E1,F1,G1) show higher magnification views of (A,B,C,D,E,F,G). Abbreviations: Ppi, parapineal organ; Pi, pineal organ; L, left; R, right. Scale bars=200µm.
