# Supplementary material for: Diversification of habenular organization and asymmetries in teleosts: Insights from the Atlantic salmon and European eel
Source: Front Cell Dev Biol. 2022 Nov 3;10:1015074. doi: 10.3389/fcell.2022.1015074 (PMC9671474; doi:10.3389/fcell.2022.1015074)
Supplement: Supplementary file 6 [file DataSheet14.PDF]

Parr Upper mode					
Individual	Section number	Left		Right	
		Ventral	Dorsal	Ventral	Dorsal
N°1	33/72	94	61	92	73
	39/72	80	60	89	61
	Σ	174	121	181	134
N°2	30/60	113	68	102	66
	33/60	114	57	99	69
	Σ	227	125	201	135
N°3	27/72	109	67	106	76
	35/72	141	65	148	83
	Σ	250	132	254	159
P-value		4.32E-02		3.82E-02	

Smolt					
Individual	Section number	Left		Right	
		Ventral	Dorsal	Ventral	Dorsal
N°1	32/70	90	48	104	55
	35/70	86	61	90	54
	Σ	176	109	194	109
N°2	37/72	100	68	95	75
	43/72	100	62	92	67
	Σ	200	130	187	142
N°3	32/60	83	51	94	50
	40/60	84	50	87	60
	Σ	167	101	181	110
P-value		3.15E-04		2.93E-02	

Supplementary Table 3. Comparison of nuclei densities between dorsal and ventral habenula territories in Atlantic salmon upper parr (upper panel) and smolt (lower panel). For each smoltification status, the number of nuclei observed within a 3700 µm2 area of Yo-Pro1 stained sections was counted (see examples of the areas taken into account in Supplementary Figure 1L. Countings were performed for 3 specimens (column 1), and on 2 sections selected in the medial part of the left and right habenulae (section numbers in column 2) per specimen. A paired Student t-test was conducted, taking the sum of counted nuclei for each territory (left, third and fourth columns; right, fifth and sixth columns), with the following null hypothesis "absence of significant difference in the number of nuclei counted in ventral versus dorsal territories". In each case, p-values (<5E-02) support the hypothesis that nuclei densities are higher in ventral territories than in dorsal ones.
