# Supplementary material for: Diversification of habenular organization and asymmetries in teleosts: Insights from the Atlantic salmon and European eel
Source: Front Cell Dev Biol. 2022 Nov 3;10:1015074. doi: 10.3389/fcell.2022.1015074 (PMC9671474; doi:10.3389/fcell.2022.1015074)
Supplement: Supplementary file 7 [file DataSheet9.PDF]

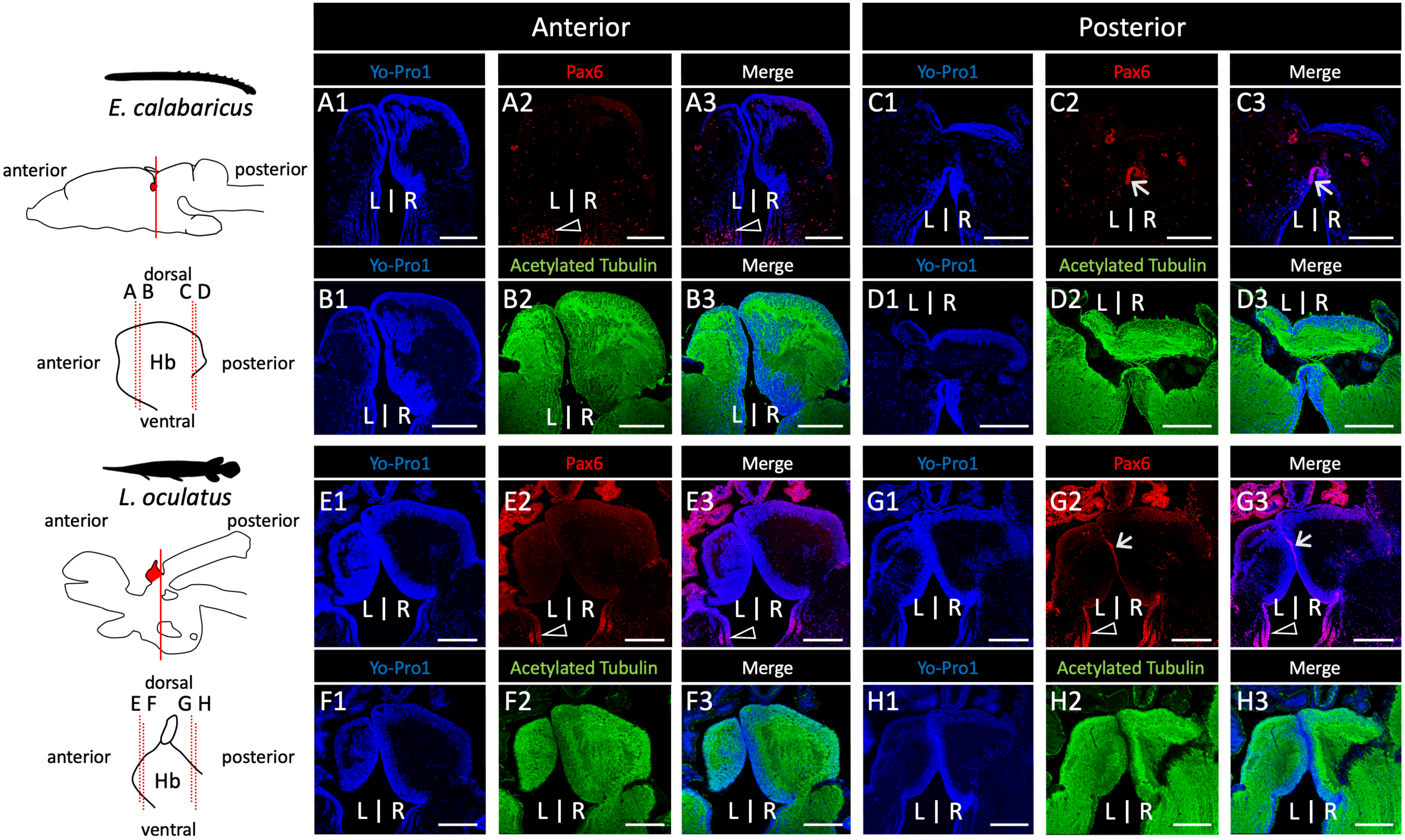

Supplementary Figure 8. No pax6 positive left dorsal nucleus is detected in the reedfish and the spotted gar. (A1-3,B1-3,C1-3,D1-3) and (E1-3,F1-3,G1-3,H1-3) show transverse sections of the habenulae, respectively in the reedfish *Erpetoichthys calabaricus* and the spotted gar *Lepisosteus oculatus*. The plane and levels of sections are shown in schematic lateral views of the brain and of the habenulae at the left of the Figure. (A-H1), (A2,C2,E2,G2) and (B2,D2,F2,H2) were respectively submitted to Yo-Pro1 staining (nuclei in blue), IHC with antibodies directed against Pax6 (red) and acetylated tubulin (green). (A3), (B3), (C3), (D3), (E3), (F3), (G3) and (H3) are respectively merged images of (A1-2), (B1-2), C1-2), (D1-2), (E1-2), (F1-2), (G1-2) and (H1-2). Opened arrowheads and thin arrows respectively point to Pax6 positive signals in the thalamus and in a ventricular territory with a pseudo-stratified neuroepithelial cell organization suggestive of neural progenitors. Vertical bars indicate the midline. L, left; R, right. Scale bars=200µm.
