# Supplementary material for: Diversification of habenular organization and asymmetries in teleosts: Insights from the Atlantic salmon and European eel
Source: Front Cell Dev Biol. 2022 Nov 3;10:1015074. doi: 10.3389/fcell.2022.1015074 (PMC9671474; doi:10.3389/fcell.2022.1015074)
Supplement: Supplementary file 8 [file DataSheet11.PDF]

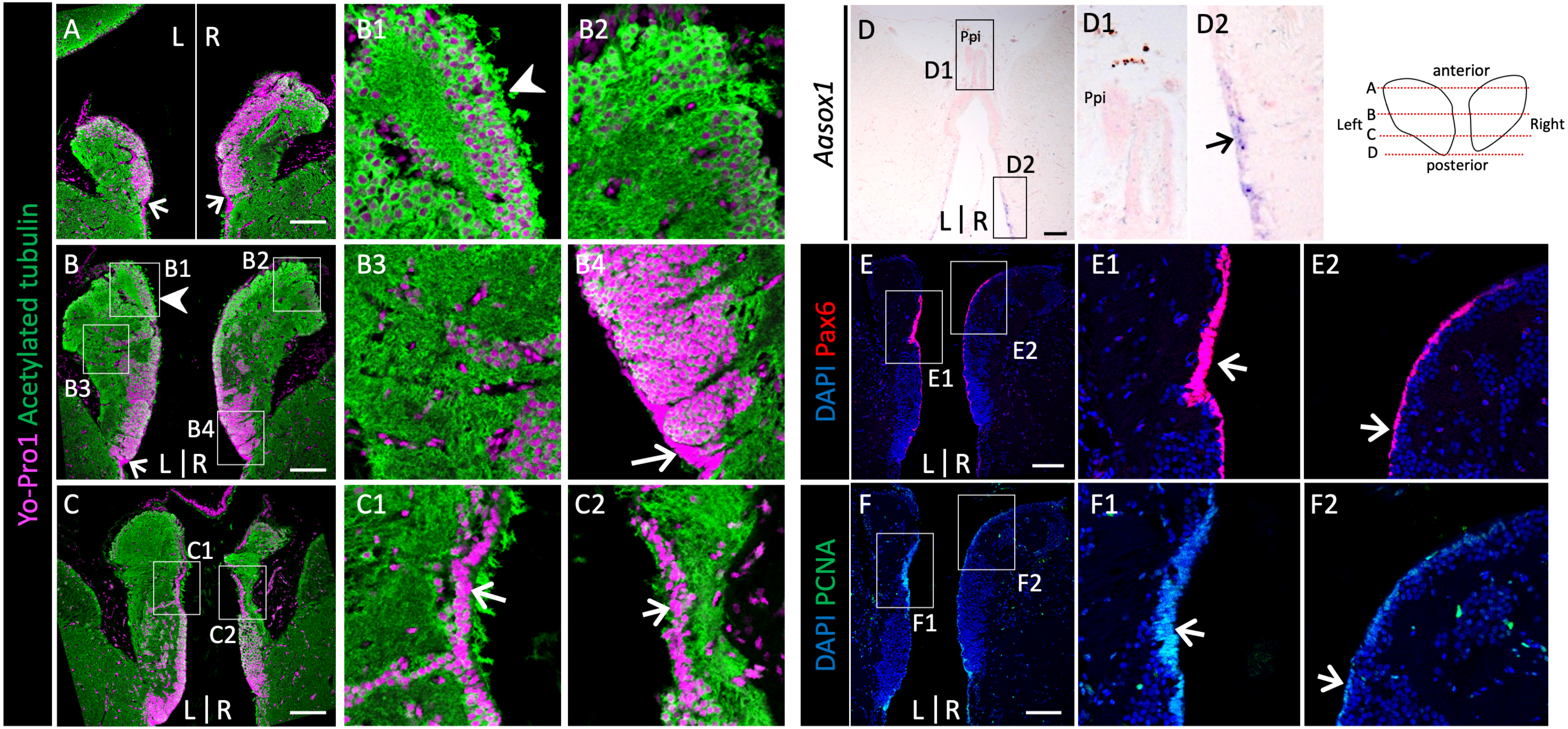

Supplementary Figure 10. IHC and ISH characterization of European eel habenulae. (A,B,C) show transverse sections of silver eel habenulae following Yo-Pro1 staining (magenta) and IHC with an antibody directed against acetylated tubulin (green). **(D)** shows a transverse section of silver eel habenula at a posterior organ level following ISH with a *sox1* probe, with a *sox1* signal at the level of neural progenitors (D2) but not the parapineal (D1). **(E,F)** show adjacent transverse sections of silver eel habenulae at a posterior level following DAPI staining (blue) and IHC with an antibody directed against pax6 (red). (E,F), (B1,B2,B3,B4), (C1,C2), (D1,D2), (E1,E2) and (F1,F2) are higher magnifications of the areas boxed in (B), (C), (D), (E) and (F) respectively. A vertical line indicates the midline. White arrows point to neural progenitors, a white arrowhead points to the left restricted, pax6 positive dorsal nucleus. L, left; R, right. Scale bars=200µm.
