# Supplementary material for: Diversification of habenular organization and asymmetries in teleosts: Insights from the Atlantic salmon and European eel
Source: Front Cell Dev Biol. 2022 Nov 3;10:1015074. doi: 10.3389/fcell.2022.1015074 (PMC9671474; doi:10.3389/fcell.2022.1015074)
Supplement: Supplementary file 9 [file DataSheet3.PDF]

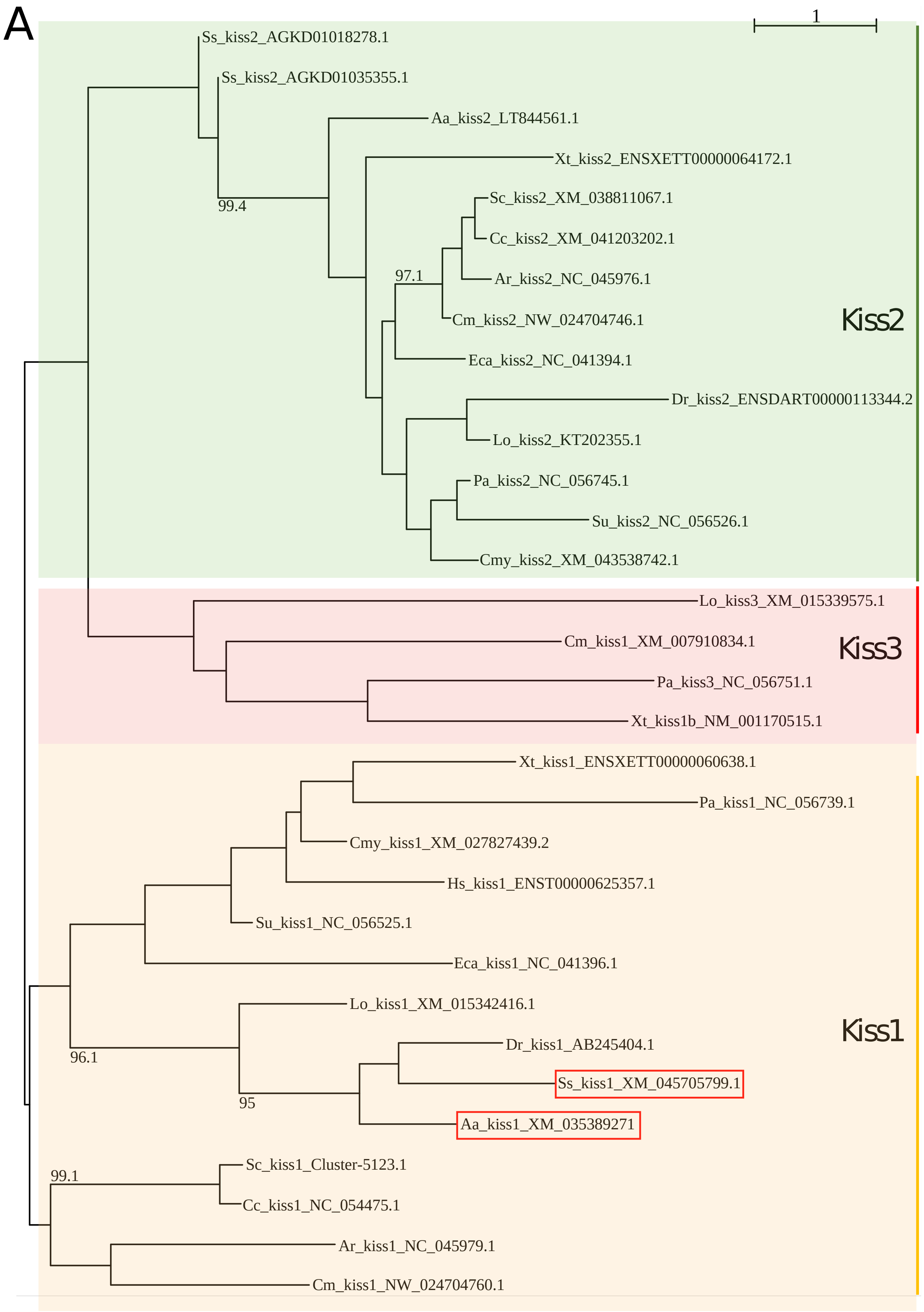

Supplementary Figure 2 (continued on next page)

B

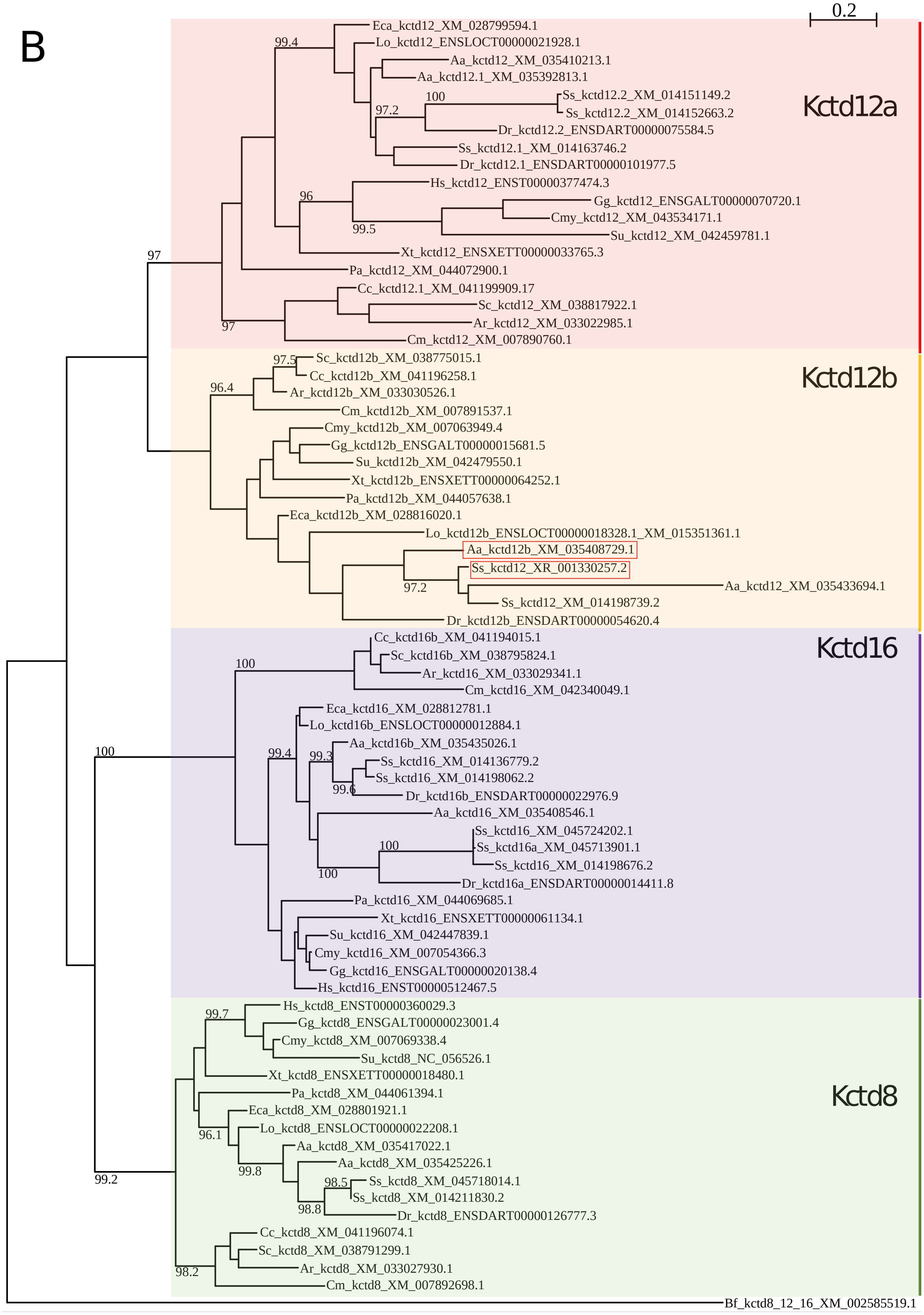

Supplementary Figure 2 (continued on next page)

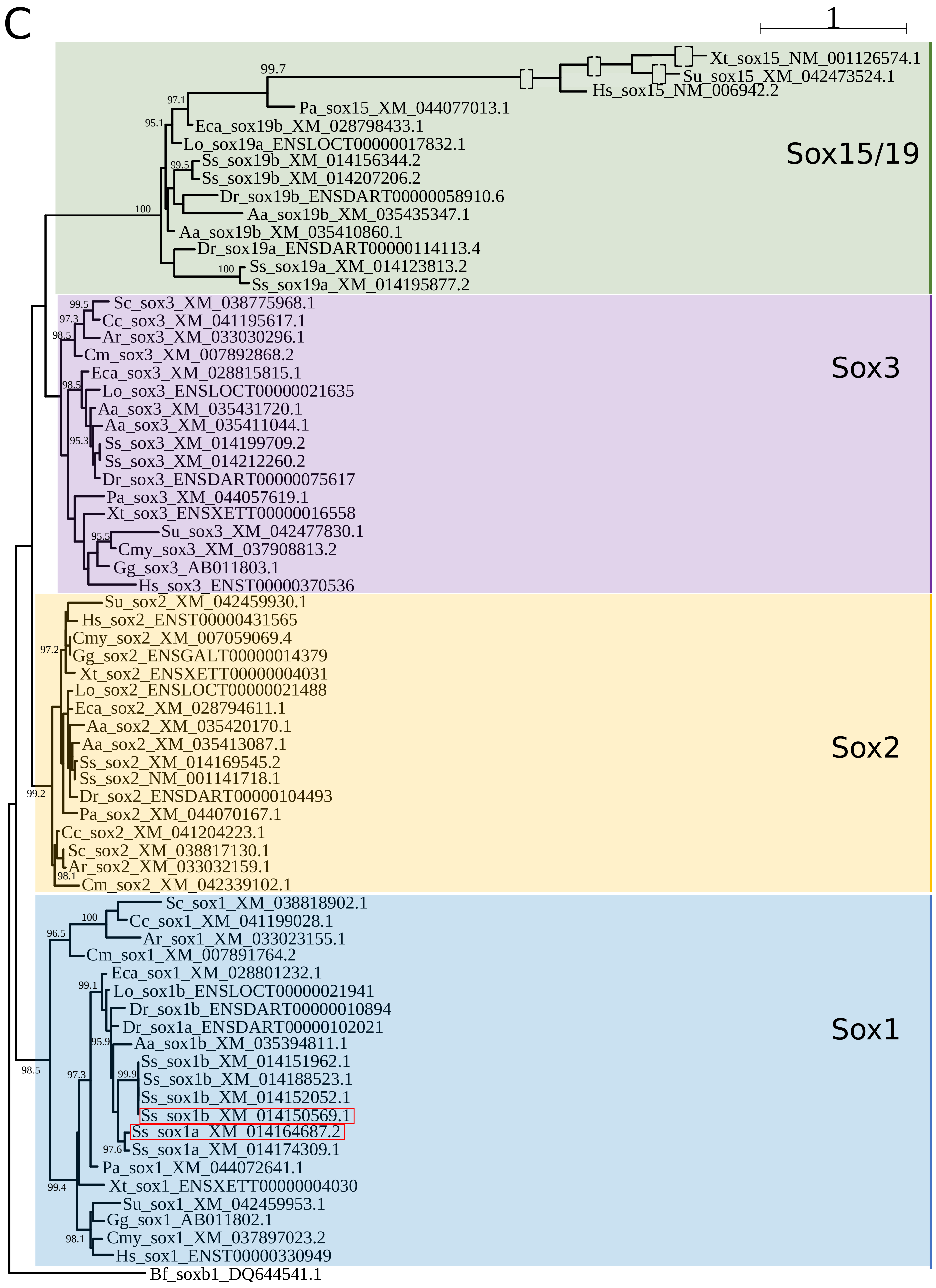

Supplementary Figure 2 (legend on next page)

Supplementary Figure 2. (A-C) ML trees showing phylogenetic relationships within gnathostome Kiss (A), Kctd8/12a/12b/16 (B) and Sox1/2/3 (C) gene families. Gnathostome orthology classes (*Kiss1*, *Kiss2* and *Kiss3*; *Kctd8*, *Kctd12a*, *Kctd12b* and *Kctd16*; *Sox1*, *Sox2*, *Sox3* and *Sox15/19)* are shaded in color. Genes are identified by their accession numbers and those analyzed by ISH in this study are boxed in red. The tree was constructed by PhyML (version 3.0), integrated into Seaview 4.2, using the Maximum Likelihood method and the LG-F+Γ12+I substitution model. SPR was used to compute the tree. Statistical supports are displayed at the corresponding nodes when higher than 0.95. Scale bars indicate the number of substitutions per position for a unit branch length, except for the branches of the highly divergent *Sox15/19* class in (C). Species names: *Aa, Anguilla anguilla; Ar, Amblyraja radiata ; Cc, Carcharodon carcharias ; Ch, Clupea harengus ; Cm, Callorhinchus milii ; Cmy, Chelonia mydas; Dr, Danio rerio; Eca, Erpetoichthys calabaricus ; Gg, Gallus gallus; Hs, Homo sapiens; Ip, Ictalurus punctatus; Lo, Lepisosteus oculatus; Mc, Megalops cyprinoides; Pa, Protopterus annectens; Sc, Scyliorhinus canicula; Sf, Scleropages formosus; Ss, Salmo salar; Su, Sceloporus undulatus ; Xt, Xenopus tropicalis.*
