# Supplementary material for: Diversification of habenular organization and asymmetries in teleosts: Insights from the Atlantic salmon and European eel
Source: Front Cell Dev Biol. 2022 Nov 3;10:1015074. doi: 10.3389/fcell.2022.1015074 (PMC9671474; doi:10.3389/fcell.2022.1015074)
Supplement: Supplementary file 10 [file DataSheet1.PDF]

Diversification of habenular organization and asymmetries in teleosts: insights from the Atlantic salmon and European eel

Supplementary Material

List of Supplementary Figures and Tables

Supplementary Figure 1. Cytoarchitecture of habenulae in Atlantic salmon.

Supplementary Figure 2. ML trees showing phylogenetic relationships within gnathostome *Kiss, Kctd8/12a/12b/16* and *Sox1/2/3* gene families.

Supplementary Figure 3. Subdomain organization of habenulae in Atlantic salmon during smoltification.

Supplementary Figure 4. 3D reconstruction of *Sskctd12b, Sskiss1* and *Sssox1b* expression territories.

Supplementary Figure 5. Expression of *Sssox1a/b* in the parapineal organ in the Atlantic salmon.

Supplementary Figure 6. Expression of pax6 in neural progenitors and in a left dorsal nucleus.

Supplementary Figure 7. Posterior restriction of neural progenitors during Atlantic salmon smoltification.

Supplementary Figure 8. No pax6 positive left dorsal nucleus is detected in the reedfish and the spotted gar.

Supplementary Figure 9. Loss of a *sox1* paralogue in the European eel A. *anguilla.*

Supplementary Figure 10. IHC and ISH characterization of European eel habenulae.

Supplementary Table 1. Antibody used.

Supplementary Table 2. Comparison of nuclei densities between dorsal and ventral habenula territories in Atlantic salmon upper parr and smolt

Supplementary Table 3. Sequence of probes used.
