# Supplementary material for: Diversification of habenular organization and asymmetries in teleosts: Insights from the Atlantic salmon and European eel
Source: Front Cell Dev Biol. 2022 Nov 3;10:1015074. doi: 10.3389/fcell.2022.1015074 (PMC9671474; doi:10.3389/fcell.2022.1015074)
Supplement: Supplementary file 11 [file DataSheet5.PDF]

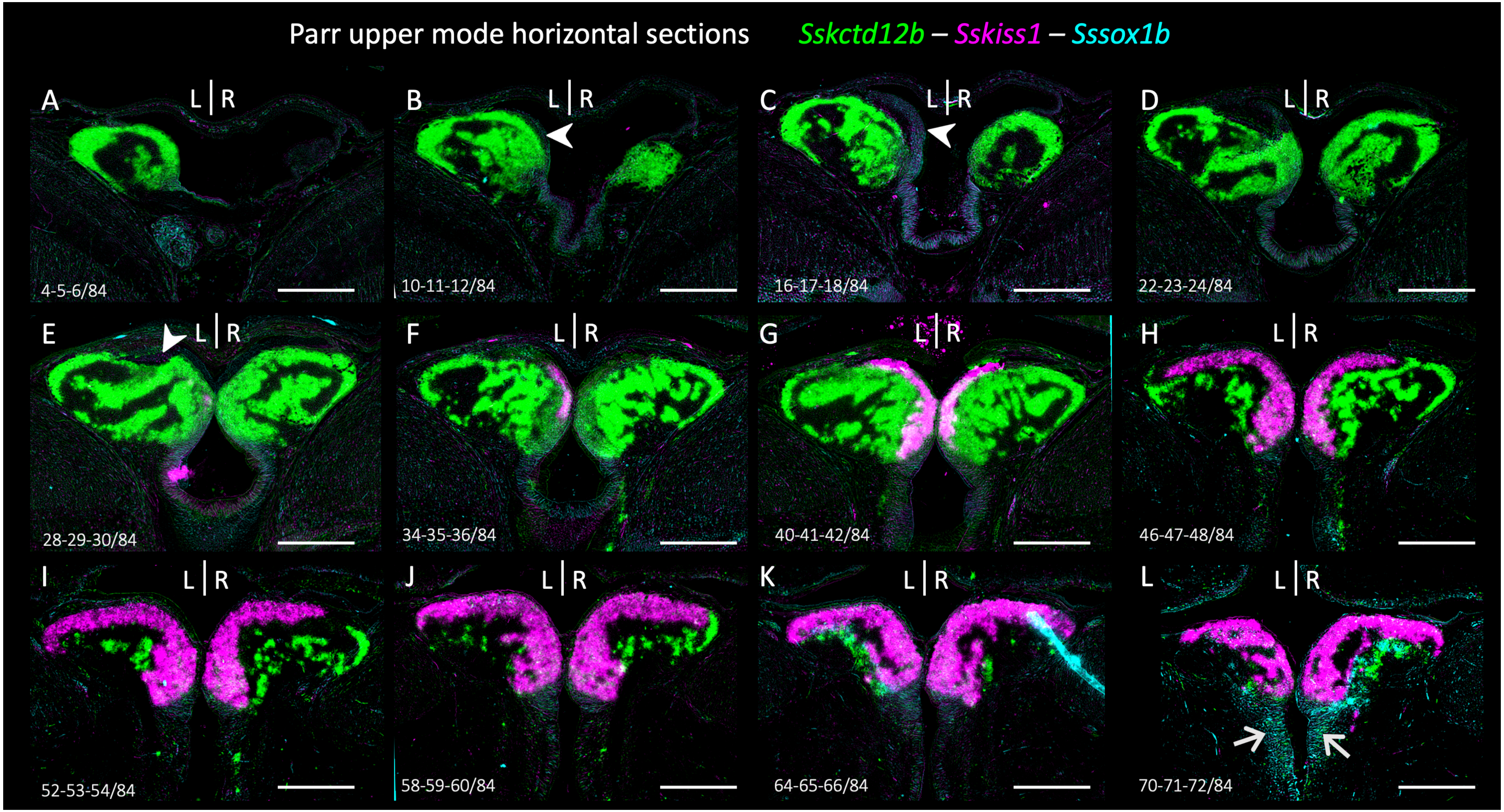

Supplementary Figure 4. 3D reconstruction of *Sskctd12b*, *Sskiss1* and *Sssox1b* expression territories. (A-L) show the result of the alignment of successive horizontal sections respectively hybridized with probes for *Sskctd12b*, *Sskiss1* and *Sssox1b* from dorsal to ventral habenula levels. Values in the bottom left corner of each panel indicate the numbers of the sections aligned. A vertical bar indicates the midline. The territories for *Sskctd12b*, *Sskiss1* and *Sssox1b* are respectively shown in green, purple and light blue. Abbreviations: L, left; R, right. Scale bars=200 µm.
