# Supplementary material for: Diversification of habenular organization and asymmetries in teleosts: Insights from the Atlantic salmon and European eel
Source: Front Cell Dev Biol. 2022 Nov 3;10:1015074. doi: 10.3389/fcell.2022.1015074 (PMC9671474; doi:10.3389/fcell.2022.1015074)
Supplement: Supplementary file 12 [file DataSheet12.PDF]

| | | |
|---|---|---|
| Anti-acetylated tubulin | Sigma T-6793 | 1:300 |
| Anti-proliferating cell nuclear antigen (PCNA) | Sigma P8825 | 1:2000 |
| Anti-proliferating cell nuclear antigen (PCNA) | Dako M0879 | 1:800 |
| Anti-Pax6 | Ozyme BLE901301 | 1:300 |

**Supplementary Table 1. Antibody used.** The last column indicates concentration used in IHC.
