# Supplementary material for: Diversification of habenular organization and asymmetries in teleosts: Insights from the Atlantic salmon and European eel
Source: Front Cell Dev Biol. 2022 Nov 3;10:1015074. doi: 10.3389/fcell.2022.1015074 (PMC9671474; doi:10.3389/fcell.2022.1015074)
Supplement: Supplementary file 13 [file DataSheet8.PDF]

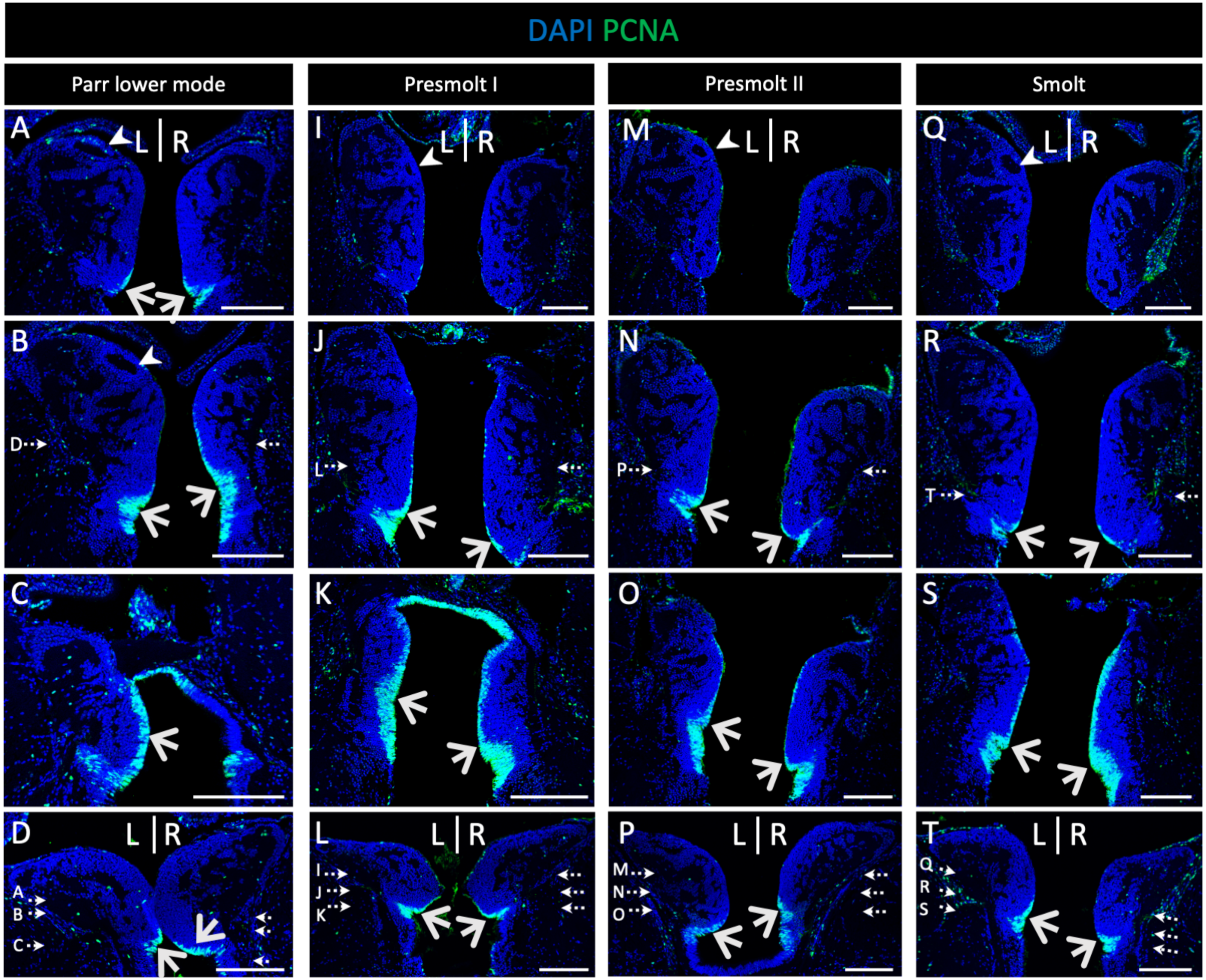

Supplementary Figure 7. Posterior restriction of neural progenitors during Atlantic salmon smoltification. (A-T) show transverse (A-C,I-K,M-O,Q-S) and horizontal (D,L,P,T) sections of Parr lower mode (A-D), pre-smolt I (I-L), pre-smolt II (M-P) and smolt (Q-T) Atlantic salmon specimens, following IHC with an antibody directed against PCNA. (A,B,C), (I,J,K), (M,N,O) and (Q,R,S) correspond to sections at anterior, medial and posterior organ levels. The level of the sections shown in (D,L,P,T) is indicated by a dotted arrow in (B,J,N,R). Vertical bars indicate the midline. White arrows point to neural progenitors, white arrowheads point to the left restricted, pax6 positive nucleus. L, left; R, right. Scale bars=200µm.
