# Supplementary material for: Diversification of habenular organization and asymmetries in teleosts: Insights from the Atlantic salmon and European eel
Source: Front Cell Dev Biol. 2022 Nov 3;10:1015074. doi: 10.3389/fcell.2022.1015074 (PMC9671474; doi:10.3389/fcell.2022.1015074)
Supplement: Supplementary file 2 [file DataSheet13.PDF]

Gene Name	Species	NCBI accession number	Salmonid duplicate	NCBI accession number salmonid duplicate	% identity probe with salmonid duplicate	Sequence
kiss1	*Salmo salar*	XM_045705799.1				ATGCAATGCTAAAGGTCTTGAGAGATTTATCCACTGCATCAACAGAGGGCCCACTCCCTGACAAACTTCCTGCTCTTCTACCCAATGAC<br>GTAGACAATCCACTGTTAGAGGCGGAACTACCCAGAAGGATGTGGTGGTGGTGGTTCCCAGCGAAGCCCCACCCACAAAACAGGATT<br>CGTCAGAATCGAGCTTGGAGGTCCTCGTACAACCATAACTCCTTTGGCCTACGTTACGGGAAATGA
sox1a1	*Salmo salar*	XM_014164687.2	*sox1a2*	XM_014174310.2	98.8	ATGCACCGCTACGACATGTCGGCGCTCCAGTACAGCCCCATCTCGAACTCTGAGGGCTACATGAGCGCCTCTCCGTCCGGCTACGGCG<br>GGATCACCTACACGCAGCACCAGAGCTCTGGCGTCTCCTCCTCGGCTGCCATGGGCACGTTAGGGTCGCTGGTGAAGTCAGAGCCGA<br>GCGTAAGCCCTCCCGTTAGCACTCACTCCAGGGGCCCTTGCCCCGGAGACTTGAGAGAGATGATAAGCATGTATTTACCGACCGGAGA<br>GCCCGGGGACCCGTCGGTGCAAAGTAGACTCCACGCA
sox1b1	*Salmo salar*	XM_014150569.2	*sox1b2*	XM_014152052.2	98.5	TATAGTATGATGATGGAGACTGACCTACACTCCCCAGTGCCCCAAACGAACACGAATTCGGGGCACACGGGAGGATCAAACAATGGG<br>AACAAAGTCAACCAAGACCGCGTAAAGAGACCGATGAACGCCTTCATGGTTTGGTCCCGGGGCCAGCGGCGGAAGATGGCTCAGGAG<br>AACCCCAAAATGCACAACTCCGAGATCAGCAAGCGACTCGGCGCCGAGTGGAAGGTGATGTCCGAGGCGGAGAAGAGACCGTTCATC<br>GACGAGGCGAAGCGGCTACGAGCTATGCACATGAAGGAGCATCCGGATTATAAATACAGACCGAGACGGAAGACCAAGACACTGTTA<br>AAAAAGGACAAATACTCTTTGGCCGGTGGGCTGCTCACCGGGACAGGGGGTGGGGGTGTCGGTCTAGGGATGAGCCCTGGTGTCGG<br>ACAAAGGTTAGACAGTCCCGGGGGTCACGGGGGTGCGTCTGCTGCCAGCTACGCGCACATGAACGGCTGGGCGAACGGTGCGTACT<br>CGGGGCAGGTGGCTGCAGCTGCGGCGGCCGCGGCCATGATGCAGGAGGCTCAGCTCGCCTACAGCCAGCACCCCGGCAGC
Kctd12b	*Salmo salar*	XR_001330257.2				GCTCTGCCAGATAACACCCCCAGCATTCCATTAGGGGAAGAAGTAATCTTCCCTGAGATAGTAGAGCTGAATGTGGGTGGCCAGGTGT<br>ACATCACTCGCTACTCAACCCTTATCAGCGTACCAGACTCTCTCCTGTGGGAGATGTTTAACAGGAAATCCACCAAAGGTCTGGCGAG<br>GGACACCAAGGGCCGTTTCTTTGTGGACCGGGATGGTTTCTTGTTCCGGTATATTCTGGACTACATGAGAGATCAGCAGCTGGTGCTC<br>CCAGACCACTTCCCAGAGCGGGGTCGCCTGCAGCGGGAGGCTGAGTTCTTCATCCTACCTGAGCTCGTAAAGATTCTGGCGCCCAAAT<br>TCAGCAAGCAGAACTCGCTAGGCGACGAGGGGTGTCAGAGCGACCCGGAGGACTCCTCGCCGGGCATTGAAACCGGGCGCAACCTG<br>GCCTCGCTGGGCACTGCCGCCTGCGCCAGTCTGGTCCCCAGTGGAGACGGTAAACGCTCGGGATTCATCACCATCGGTTATCGCGGC<br>TCTTATACATTGGGGCGCGACAGCCAGACCGATGCCAAGTTCAGACGTGTTGCTCGCATTATGGTCTGTGGCAAG
kiss1	*Anguilla anguilla*	XM_035389271				ATGACAGACATGCTGTGGGCCAGATCTGCCCCAGCTCCCCTCTCCCATGCCACCATTCTGCTGTTGACCGTGGTCTCCATGGTGACCAT<br>CAGGTTTGGGGAGACCAGGTCTCTTAGGTACACCCCGTACACTGCAGATGAAGACCCAGAGATTGCAGCCATGCGGGTCCTGAGGCA<br>AATGAACGAGGAGCTGTCGACCGAGGCCCCGCCCTCCCCGCACCGCCCCGCCCACTGGCCCACAGCACTGGCCAATCCACTGCCGGG<br>AGGGGCCGGCCTTCCCAGGAGGTCGTGGTGGTGGTACCCCGAGGTGCCGCCACAACAGGCCAAGAAGCGGGAAAACTTCTCCTCCTA<br>CAACTGGAACTCTTTCGGTTTACGTTATGGGAAATGA
sox1	*Anguilla anguilla*	XM_035394811.1				ATGTATAGCATGATGATGGAAACGGACCTGCACTCCCCAGGACCGCAAACCAATACAAATTCGGGCCAAACGTTGCCAAACAACGGGA<br>GCAAAGCTAATCAAGACCGAGTAAAAAGACCTATGAATGCATTTATGGTTTGGTCTCGGGGCCAGCGGAGGAAGATGGCACAGGAGA<br>ACCCCAAAATGCACAACTCTGAAATCAGTAAGAGGCTGGGTGCGGAGTGGAAAGTTATGTCTGAGGCGGAAAAGAGACCCTTTATCG<br>ATGAGGCAAAGCGGTTACGGGCCATGCATATGAAAGAACATCCGGATTACAAGTACAGACCCAGGAGAAAGACCAAGACGCTGCTCA<br>AAAAAGACAAATACTCTTTAGCCGGCGGTTTGCTAACCGGGGGTGGCAGCGGAGGAGGTGTTGGTCTTGGGGTCGGGGTGAGTCTAG<br>CAGGAGTCGGACAGAGGTTAGACAGCCCTGGTAGTCACGGAGGCACAGCTAGTACCGGCTATGCACACTTAAACGGCTGGGCAAACG<br>GCGCCTATTCGGGCCAGGTGGCGGCGGCAGCGGCGATGATGCAAGAAGCGCATATAGCTTACAACCAGCACCCCGGCAGCGGGGCG<br>CACCATCACCACGCGCACCACCCACACAAC
Kctd12b	*Anguilla anguilla*	XM_035408729.1				GCCATGAAGGAGACCATTCTGCCCATCAGCGAGGTGTCTGGCCCGGCCCCGTTGCCCTACCCGGAGGTAGTGGAGCTCAACGTGGGC<br>GGCCAGGTGTACGTCACGAAGCGCTCCACCCTGGTCAGTGTGCCCGACACCACCCTGCACGCCATGTTCACCCGGTGCCCGCCACGG<br>GAGCTACCAAGGGACAGCCGCGGGCGCTTCTTTATCGACCGCGATGGCTTCCTGTTCCGCTACGTGCTGGACTTCCTGCGAGACCGG<br>CAGCTGGTGCTGCCCGACCACTTCCCTGAACGTGAGCGCCTGCAGAGGGAAGCCGAGCACTTCCAGCTGGGCGAACTGCTGCGCCTC<br>CTGGGGCCCCGGGTGGGTTCCAAGCAGGGCTCCATGAATGATGAAGGCTGCCACAGTGATATTGAGGAGAGCTCACAAAGCAGTGAG<br>TACCCCACATGTCGTACCTTGACTTCCTCGGACAAGAGGTCAGGGTTCATCACCATTGGCTACAGGGGATCCTACACCACGGTGCGGG<br>ACAACCAAGCCGATGCCAAGTTCCGACGCGTGGCCAGGATCATGGTGTGCGGACGCATCGCCCTGGCCAAAGAGGTG

Supplementary Table 2. Sequence of probes used. In view of the similarity level of the probe For *Sssox1a1* and *Sssox1b1*, cross-hybridization with the sequences of paralogues generated by the salmonid duplication (respectively *Sssox1a2* and *Sssox1b2*) cannot be excluded. We therefore refer to *Sssox1a* and *Sssox1b* for the corresponding profiles in the text.
