# Supplementary material for: Diversification of habenular organization and asymmetries in teleosts: Insights from the Atlantic salmon and European eel
Source: Front Cell Dev Biol. 2022 Nov 3;10:1015074. doi: 10.3389/fcell.2022.1015074 (PMC9671474; doi:10.3389/fcell.2022.1015074)
Supplement: Supplementary file 14 [file DataSheet10.pdf]

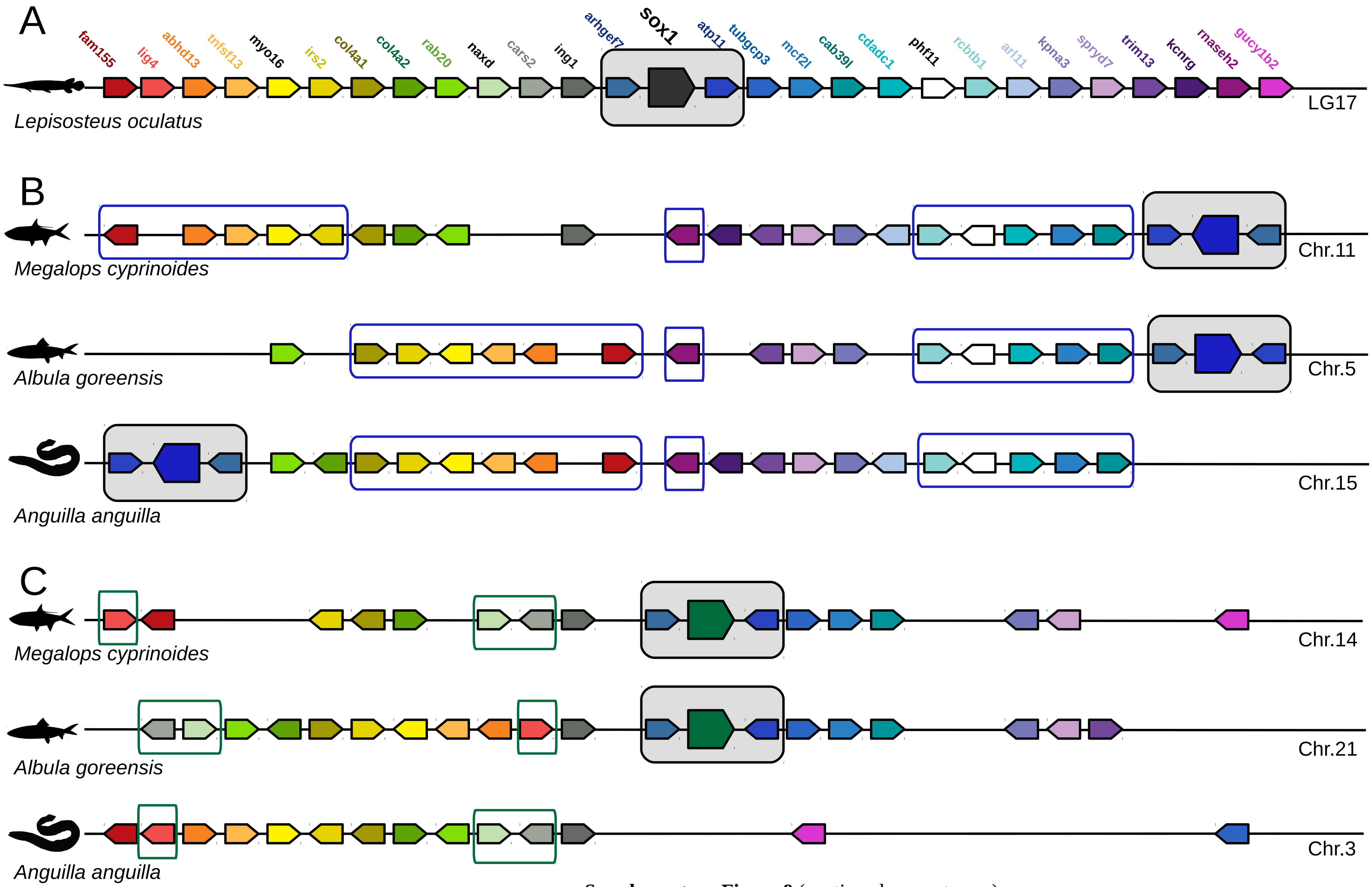

Supplementary Figure 9 (continued on next page)

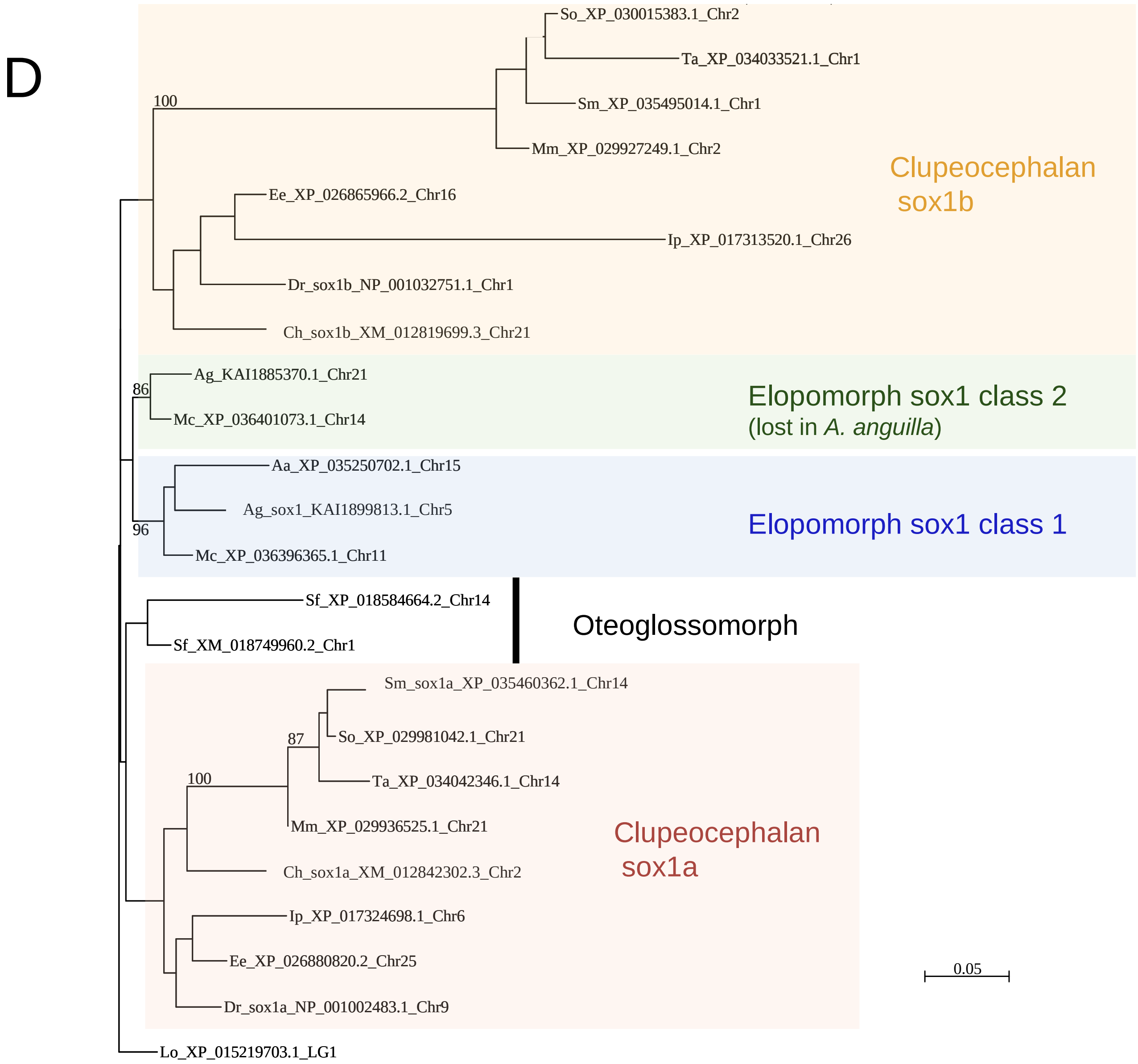

Supplementary Figure 9. Loss of a *sox1* paralogue in the European eel A. *anguilla.* (A,B,C) Conserved syntenies in the vicinity of *sox1* between the spotted gar (A) and three elopomorphs, *M. cyprinoides*, *A. goreensis* and *A. anguilla* (B,C). Genes are shown as colored arrowheads, names corresponding to colors as indicated in (A). Arrowhead orientations correspond to those of genes on the (+) strand of each chromosome, except for *Albula goreensis* chromosome 5, where they indicate those on the (-) strand. Two paralogous loci, respectively shown in (B) and (C), are identified in the three elopomorphs analyzed, in agreement with the 3R whole genome duplication of teleosts. One of these loci, shown in (B), contains a *sox1* representative (symbolized by a large blue arrowhead) in all three elopomorphs. The other one, shown in (C), has undergone a deletion including the gene in the European eel (but not in *M. cyprinoides* and *A. goreensis*, in which the *sox1* gene is symbolized by a green arrowhead). A conserved synteny in the immediate vicinity of *sox1*is shown by a gray box. Blue (B) and green (C) boxes show genes or gene linkages selectively conserved in either one of the two paralogous loci. **(D)** ML tree showing phylogenetic relationships between *sox1* genes of teleosts. The *sox1a* and *sox1b* orthology classes of clupeocephalans are shown in color but their relationships with the two classes observed in elopomorphs and in *Scleropages formosus* remain unresolved. Genes are identified by their accession numbers. The tree was constructed by PhyML (version 3.0), integrated into Seaview 4.2, using the Maximum Likelihood method and the LG-F+Γ12+I substitution model. SPR was used to compute the tree. Statistical supports (in %) are displayed at the corresponding nodes when higher than 0.85. Scale bars indicate the number of substitutions per position for a unit branch length. Species names: *Aa*, *Anguilla anguilla*; *Ag*, *Albula goreensis*; *Ch*, *Clupea harengus*; *Dr*, *Danio rerio*; *Ee*, *Electrophorus electricus*; *Ip*, *Ictalurus punctatus*; *Lo*, *Lepisosteus oculatus*; *Mc*, *Megalops cyprinoides*; *Mm*, *Myripristis murdjan*; *Sf*, *Scleropages formosus*; *Sm*, *Scophthalmus maximus*; *So*, *Sphaeramia orbicularis*; *Ta*, *Thalassophryne amazonica*. Abbreviation: Chr, chromosome.
